# Balancing cash and food: The impacts of agrarian change on rural land use and wellbeing in Northern Laos

**DOI:** 10.1371/journal.pone.0209166

**Published:** 2018-12-31

**Authors:** Puwadej Thanichanon, Dietrich Schmidt-Vogt, Michael Epprecht, Andreas Heinimann, Urs Wiesmann

**Affiliations:** 1 Department of Geography, Ramkhamhaeng University, Bangkok, Thailand; 2 Faculty of Environment and Natural Resources, Freiburg University, Freiburg, Germany; 3 Centre for Development and Environment (CDE), University of Bern, Bern, Switzerland; University of Manitoba, CANADA

## Abstract

This study investigates the effects of improved market accessibility on agricultural land use and basic wellbeing, defined by income and rice sufficiency, in Xayaburi province, Lao PDR through a meso-scale and actor-oriented approach with data collection at both district and household level. It also investigates farmers’ decision-making as it relates to regional markets. Increasing market accessibility in rural areas facilitates cash crop trade leading to agrarian change from subsistence to commercial agricultural systems. This transformation raises concerns about food security and vulnerability to market uncertainties as farmers are likely to grow cash crops intensively and in place of food crops, leading to lower food production. Meanwhile incomes from cash crop trade are highly vulnerable to market uncertainties. We found that farmers in the south of Xayaburi, where market accessibility is higher than in the north, primarily grow cash crops and do not suffer from rice insufficiency while farmers in the north, where market accessibility is lower, rely more on subsistence agriculture and have a lower level of basic wellbeing. The major factors of better basic wellbeing in the south include: (1) better market accessibility which can mitigate the risks of market uncertainty and create enough income to compensate for and overcome losses in rice production, (2) availability of more arable land due to a larger amount of level terrain which allows farmers to expand cash crop cultivation and continue growing rice at the same time, and (3) farmer strategy to keep a part of their land for growing rice to meet their minimum consumption needs and prevent the risks of rice insufficiency.

## Introduction

Rural areas in developing countries are often prone to poverty [1–3]. Rural poverty results mainly from low potential for agriculture, poor infrastructure, weak market institutions and political isolation [4]. These factors are associated with remoteness and weak integration into markets leading to limited market access and low income from farm produce [4–6]. Limited access to markets is thus one of the core determinants of rural poverty.

Market creation and infrastructure development in rural areas have been encouraged based on the consideration that these measures can mitigate rural isolation, create channels for income generation, and expand people’s access to services [4,5,7–10]. Improved access to agricultural markets can play an important role in rural development as it integrates rural areas into urban markets, and creates new trade routes and commodity chains [7]. Improved accessibility enables flows of goods, people, resources and information [11]. Integrated markets are markets in which the prices of commensurable goods do not behave independently [12] which leads to the unimpeded flow of commodities, low price fluctuation and better access to the products [13]. Greater competition in integrated markets drives down consumer price, making goods affordable and thus increasing the access of households to markets. Better access to integrated markets stimulates demand and increases trading opportunities [13]. Improvement of market accessibility is also expected to diversify economic activities and increase incomes for people in rural areas [4,13] where traditional subsistence production systems are considered vulnerable, less diverse, and typically generating only low income [6,9]. Traditional cultivation systems are, moreover, vulnerable to natural disasters [13]. Improved market integration can reduce these risks and even contribute to conservation by lowering dependency on natural resources [14].

Market accessibility improvement and better market integration have brought about in many developing countries rural agrarian change from subsistence to market-oriented systems [10,15–18]. Improvements in access to capital and services such as credit, technology, agricultural inputs, information and irrigation, often occur alongside improved market access, and facilitate local peoples’ entrance into cash crop cultivation or other commercial occupations [6,13,19]. Drivers of agrarian change related to market accessibility improvement also include an increase of demand for specific crops, infrastructure development and government policies supporting agricultural commercialization. While demand for agricultural crops was in the past mainly driven by domestic markets, cross-border demand has become more important as a result of globalization: the emphasis on the expansion of the home market that prevailed during the mid-twentieth century has been largely, but not completely, replaced by an emphasis on the promotion of an agricultural export-led strategy [20].

The shift of an agrarian system towards market-orientation is usually coupled with a shift to intensive cash crop production, and often monoculture production. Annual cash crops are cultivated intensively with the use of machines, chemical fertilizer, hybrid seeds and other farming technologies to increase productivity and sell products in large quantities [10,21,22]. Crops that are demanded by markets replace traditional crops that are mainly grown for household consumption. The boom of cash crop intensification can be seen, for example, in the rise of rice intensification in lowland Southeast Asia [21], maize and sugar cane in Laos [23], maize and cassava in Vietnam [10], maize in Tanzania [24], and maize and tobacco in Malawi [25]. Besides annual crops, perennial trees such as rubber and eucalyptus are also grown for commercial purposes as is the case in Southeast Asia [21,26] and India [27]. Commercialization also often leads to specialization in a small number of marketable cash crops usually grown in monocultures [5,21,23,28–33]. The tendency towards mono-cropping is reinforced by contract farming and concessions which compel farmers to grow only contracted crops [30,34].

Intensification of agricultural systems often results in better productivity and higher income for local farmers [33–35] as has been shown by studies in Thailand [36], Vietnam [10], Philippines [37], India [28], Malawi [25], Nigeria [17] and Guatemala [38].

However, agrarian change as a consequence of export-oriented production of cash crops also raises the concern of potentially negative impacts on basic wellbeing. Borras [39] cautioned that “…while there had been dramatic increases of cross-border agricultural trade in the last few decades, the impacts in terms of food security, household incomes and inequality have been varied and uneven”. A major concern is food security, as cash crops replace subsistence staple crops, resulting in a decline in food production for household consumption [29]. Studies of contract farming in Africa and Asia reaffirm that decreasing food availability can be an issue [10,34]. Many farmers who for various reasons are not able to adjust their practice along the agrarian change trajectory towards intensification, sometimes lose their land to agribusiness or face other problems of access to land for food production, and thus encounter food insecurity [40].

The study of food security has expanded beyond the immediate concern of rice or other staple crop sufficiency to include nutritional status [41]. In this context, diversity in the rural agricultural landscape is regarded as a prerequisite for supporting a diverse and healthy diet [40]. Agrarian change towards commercialization in the form of large-scale establishment of plantations of only one or a few cash crops can, therefore, not only reduce the quantity of food produced, creating a risk of food insecurity, but can also negatively impact the diversity of food and the quality of nutrition [42,43]. Although we are aware of the importance of nutrition, we limit ourselves to rice sufficiency as the main indicator for basic wellbeing as we are lacking data on nutritional status. Although income from selling cash crops is supposed to be high enough to buy food from the markets as a substitute for food no longer produced on the farm, cash crop specialization, as found in many studies, can be susceptible to income loss from crop failure, price fluctuation, inefficient market institutions and exploitation by buyers [29,30,34,38,44,45], thus reducing the purchasing power of farmers. In addition, food security is also affected by market accessibility. While better access to food markets can increase food security [46], some cases such as in Mozambique [46] and Tanzania [47] reveal that the negative effects of increasing food prices can counteract the benefits of better market accessibility and affect the basic wellbeing of rural dwellers.

Wellbeing is composed of several dimensions–material wellbeing, bodily wellbeing, social wellbeing, security and freedom of choice and action [1]. Basic wellbeing is mainly assessed through material assets and the availability of public services [48,49]. Income and the satisfaction of food and other basic needs are often used as indicators of basic wellbeing. The dimensions of social wellbeing such as freedom are not often used as major measures of wellbeing, particularly at aggregate levels, mainly because they are difficult to measure but also because of their unclear link with material welfare [2,3]. Wellbeing in this study thus is defined as basic wellbeing determined through income and rice sufficiency.

This study investigates agricultural practice and farmers’ wellbeing, focusing on income and rice sufficiency in the context of agrarian change towards commercialization. The study site is Xayaburi province in northern Laos where agrarian change is stimulated by cross-border trade with Thailand. The study site is characterized by a gradient of lower market accessibility in the north to higher accessibility in the south. The objectives of the study are (1) to analyze the processes of change in agricultural practice and basic wellbeing in a context of improved market accessibility, and (2) to investigate the factors that determine the balance between income generation and rice sufficiency as a means to improve basic wellbeing for the long-term.

## Methods

This study applied two approaches: the meso-scale approach and the actor-oriented approach. The meso-scale approach was used to analyze the complex interactions between market access, agricultural practice and basic wellbeing. There is a challenge of disconnect between the large scale or regional scale which permits the analysis of quantitative data and statistics, and the local scale for which information is generated through case studies [50]. A study on the regional level alone, though able to detect changes more accurately, may not be able to fully identify the processes or causes of change. On the other hand, local contexts alone cannot provide the larger picture of regional dynamics of natural resource use and change. The meso-scale approach has therefore been developed to generate and synthesize knowledge at an intermediate level between the regional level and the local level. It has the advantage of linking generalizations at a higher level with the heterogeneity of different development contexts at the local level [51].

The study also employed the actor-oriented approach, focusing on local farmers This approach helps to understand how regional markets influence farmers’ decision-making in agricultural land use, and thus provides a link between the regional and the local level [52].

We collected data about market accessibility, agricultural practice, and basic wellbeing in the study area. Although we are aware of the importance of other aspects of wellbeing as stated in the introduction, the study regarded income and rice sufficiency as main indicators of wellbeing which is in line with income and food security being the most widely used indicators to determine livelihood or poverty standards [4,7,9]. Another reason for selecting income and rice sufficiency as indicators of basic wellbeing was data availability. Rice sufficiency in this study is defined as the ability to access rice for consumption to meet, at least, the standard of the Lao National rice security, which is 184 kg / person / year (according to Paklai DPI poverty report 2005). Rice sufficiency was determined by rice production and by households’ strategies to access rice such as the retention degree of produced rice or the spending for buying rice. Crop composition and areas covered by specific crops are primarily indicators for agricultural practice. Accessibility to agricultural markets of a district was determined by proximity to markets, distribution of markets, road conditions, value of agricultural products for export, farmers’ incomes from agricultural products, prices of agricultural products and percentage of cash crop farmers.

The data was investigated at both the regional (district) and local (household) level using both quantitative and qualitative analyses. The data at the district level was analyzed to assess and compare the degree of market accessibility, agricultural practice and basic wellbeing between locations at the district level. Data at the village level was collected to support the analysis of the regional level. The data at the household level was analyzed to assess the effects of market accessibility on household agricultural practice and basic wellbeing. The data from both levels was then synthesized for an understanding of the impacts of access to markets on farmers’ land use and basic wellbeing across the whole study area. This involved the integration of regional and local data to explain the changes in agricultural practice and basic wellbeing driven by market integration via both quantitative and qualitative evidence derived from local actors such as farmers.

Data on market, agriculture and basic wellbeing at the district and village level was mostly secondary data collected from various sources, particularly district government reports and records from the District office of Planning and Investment (DPI), District of Agriculture and Forestry (DAFO) and District Office of Industry and Commerce (DOIC). The names of secondary data sets and of the exact sources are provided in S1 File. Data was then entered into a specifically created database to be used for spatial and statistical analysis.

Data at the household level was collected through a household survey from 15 sample villages. The villages were selected according to the following criteria; distance to towns, to major and minor border crossings, to the Mekong River, and to the main road; geographical distribution of villages; elevation and landform. Location and characteristics of these sample villages are provided in S6 File.

In each sample village, around 8 households were selected through stratified random sampling according to wealth classes (rich, medium and poor). In general, 2 rich, 4 medium and 2 poor households were selected per village. The wealth status of a household was determined by physical assets such as house, livestock, vehicle and income according to information obtained from village authorities and through researcher observation. Wealth classes were found to vary from village to village in the sense that, for example, a medium household in a rich village could be richer than a rich household in a poor village.

The total household samples are 121 households from 15 sample villages. As we focused on the differences in market access, land use activities and basic wellbeing between rich, medium and poor households, and not between villages, the sample size can be considered adequate.

Household data, both quantitative and qualitative, were collected through semi-structured interviews during the period from November 2011 to May 2012. Interviewees were asked to discuss their production and the sale of their produces, socio-economic status, problems about the agricultural activities, opinions about crop market in the future, changing livelihoods and market behavior as well as livelihood strategy face-to-face with the researcher. The household interview form is in the S2 File. The interviewees were informed by the researcher that their personal data would not be revealed and that the data would be analyzed anonymously. A written informed consent was not obtained. Conducting interviews was approved by the Centre of Development and Environment (CDE), University of Bern and by the Ministry of Agriculture and Forestry (MAF) of the Lao Government without the requirement for Ethics Approval as the Helsinki declaration does not apply to our research on land use and wellbeing. Moreover, the data was anonymized and it is not possible to track the information back to single specific households.

Data was also collected through physical surveys and village key informant interviews. GIS was the main tool for spatial analysis.

The data from both district and household level are presented in tables and explained in the results section. For reasons that will become apparent later in this paper, this study differentiates between the north and the south of the study area in terms of the impact of agrarian change. The northern part of the study area comprises 3 districts, namely Xayaburi, Xaysathan and Phiang, while the southern part comprises 4 districts, namely Paklai, Thongmixai, Kentao and Boten. The tables at regional level (i.e. Tables 1,2 and 4) are presented by district, whereas the tables at the local level (i.e. Tables 3 and 5) are divided by north and south. Of the sample households from 15 sample villages, 65 households in 8 villages are located in the south and 56 households of 7 villages are from the north.

**Table 1:** Comparison of accessibility to agricultural markets at the district level.

Districts	Percentage of villages that can be accessed by car	Number of trading companies *	Number of marketplaces **	Value of export crop products from trading companies (million THB)	Average income from cash crops per household (THB/year)	Percentage of cash crop farmers **	Average farm gate price of maize (THB/kg)
Xayaburi (n)	76	41	6	144	9,211	54	3.8
Xaysathan (n)	6	0	0	0	614	66	n/a
Phiang (n)	98	10	11	34	10,564	78	4.2
Paklai (s)	87	52	15	536	55,690	84	5.0
Thongmixai (s)	100	22	1	26	34,352	92	5.0
Kentao (s)	100	42	8	799	62,542	93	6.0
Boten (s)	100	17	7	24	21,023	86	5.0

**Sources:** researcher’s survey (2011–2012) and

* DOICs (2011)

** DPI (2008)

**Note:** (n) indicates districts in the north, (s) indicates districts in the south

**Table 2:** Agricultural land use at the district level.

Districts	Agri. area (ha)	Cash crop area (ha)	Percentage of cash crop area (%)	Avg. crop area per hh. (ha)	Avg. paddy area per hh. (ha)
Xayaburi (n)	14,424	5,499	39	0.78	0.32
Xaysathan (n)	1,657	51	3	0.86	0.02
Phiang (n)	11,154	4,213	38	0.66	0.52
Paklai (s)	33,182	25,300	76	2.05	0.44
Thongmixai (s)	4,128	2,758	67	1.51	0.68
Kentao (s)	25,584	18,949	74	2.48	0.56
Boten (s)	8,393	3,767	45	1.17	0.79
**Region**	**98,322**	**60,537**	**62**	**1.42**	**0.46**

**Source:** DPIs (2008)

**Note:** (n) indicates districts in the north, (s) indicates districts in the south

**Table 3:** Agricultural land use at the household level.

Region	No. of samples	Avg. agri. area (ha)	Avg. cash crop area (ha)	Avg. paddy area (ha)	Avg. upland rice area (ha)	Percentage of cash crop area	Percentage of food crop area
North	56	2.7 (2.0)	1.7 (1.7)	0.6 (0.8)	0.4 (0.6)	62	38
South	65	4.0 (2.8)	3.2 (2.5)	0.7 (0.6)	0.1 (0.3)	80	20
**Regional average**	**121**	**3.4 (2.5)**	**2.5 (2.3)**	**0.7 (0.7)**	**0.2 (0.5)**	**73**	**27**

**Source:** Researcher’s survey (2012)

**Note:** The value of standard deviation is provided in the brackets of each cell

**Table 4:** Income and rice production at the district level.

Districts	Avg. total income per person (THB/year)	Avg. income from cash crops per person (THB/year)	Per. of income from cash crops (%)	Total rice production (tons/year)	Rice production per person (kg/year)	Per. of poor hhs. (%)
Xayaburi (n)	33,064	9,258	28	20,668	316	6
Xaysathan (n)	19,960	7,385	37	2,670	216	17
Phiang (n)	31,292	9,075	29	22,883	440	6
Paklai (s)	38,924	20,630	53	18,280	280	1
Thongmixai (s)	30,996	18,598	60	5,127	588	1
Kentao (s)	39,824	19,912	50	16,833	432	0
Boten (s)	39,868	16,745	42	8,024	450	1
**Regional average**	**35,024**	**14,360**	**41**	**94,485**	**362**	**4**

**Source:** DPIs (2008)

**Note:** (n) indicates districts in the north, (s) indicates districts in the south

**Table 5:** Income and rice production at the household level.

Region	Avg. total income (THB/year)	Avg. income from cash crops (THB/year)	Per. of income from cash crops (%)	Avg. rice production (tons/year)
North	86,000	31,000	46	2.7
South	116,000	87,000	76	2.3
**Regional average**	**107,000**	**61,000**	**62**	**2.5**

**Source:** Researcher’s survey (2012)

Statistical analysis was carried out to examine the correlation between variables of agricultural land use and variables of basic wellbeing. Statistical analysis of the regional level was done per village unit through Pearson’s Correlation Coefficient for all 311 villages in the study area while statistical analysis of the local level was done per household unit through Spearman’s Correlation Coefficient for all 121 sample households. Cross tabulation between the variables of agricultural area and rice production was also used to analyze the correlation between these variables.

There are limitations to linking the regional and local levels or to comparing the regional with the local level, as data from the 2 levels are from different periods. Data at the regional level is from 2008 while data at the local level is from 2011–2012. The consequences of these limitations for interpretation and analysis are discussed in the results and discussion sections.

## Results

### The study area: General characteristics and market accessibility

**Fig 1:**
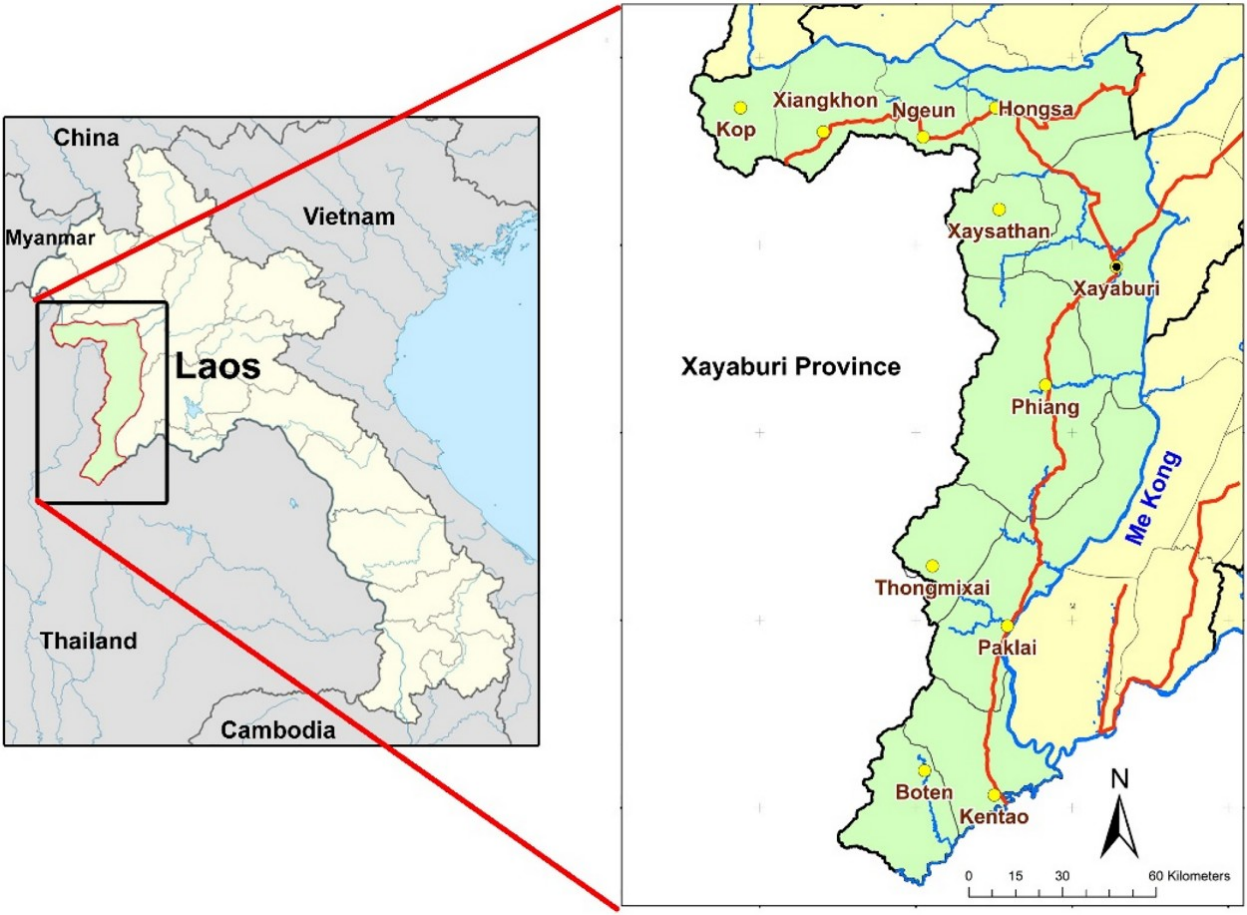
Location of Xayaburi Province, Lao PDR. The figure is based on public domain data from Openstreet map and was produced on QGIS 2.16.

The study area consists of seven districts in the middle and southern parts of Xayaburi province in the northwest of the Lao PDR (see Fig 1). In the past 10 years, Northern Laos has been a region transitioning towards a market-oriented economy [32]. Intensified regional trade with neighboring countries and the implementation of government policies of land allocation and agricultural intensification have driven agricultural transformation from subsistence agriculture to commercial agriculture, integration of markets, and development of the rural non-farming sector [13,16,18,23,32]. Traditional upland rice shifting cultivation has been largely replaced by commercial agriculture for cash crops such as maize, rubber and paddy rice.

The seven districts of the study area include Xayaburi, Xaysathan, Phiang, Paklai, Thongmixai, Kentao and Boten. The total area of the study site is approximately 11,385 km^2^. Lower elevation areas are more extensive in the south and are used widely for agriculture while the northwest of the study area is mountainous with steep slopes and an elevation over 800 meters above sea level. Most of the forested area consists of mixed deciduous forests and forest regrowth (known as unstocked forests). While the Luang Prabang Mountain Ridge in the west creates a natural boundary with Thailand, in the east, the Mekong River proves an obstacle to transportation to other provinces. Water resources in this area are abundant with several rivers flowing all year round. The climate of the study area is characterized by two seasons: the wet season from April to October (7 months) and the dry season from November to March (5 months). The average temperature is around 25°C with the average minimum 17°C in January and average maximum 28°C in August. The temperatures are lower in the northern part, especially in Xaysathan district, while the southern part is warmer and more suitable for tropical crops and livestock.

The total population of the study area in 2009 was approximately 260,690 people and 49,601 households. Xayaburi and Paklai support a larger population than the other districts. The most densely populated areas are Xayaburi town in Xayaburi and Meung Mor town in Kentao. The ethnicity in the study area is more diverse in the north, where ethnic Lao constitute around 60% of the total population, while the south is fully dominated by ethnic Lao up to nearly 100%. The study site extends in a north-south direction which is followed by National Highway 4, which is the major transportation channel. The four districts which the road passes through, Xayaburi, Phiang, Paklai and Kentao, are usually regarded, for demographic and economic reasons, as major districts of the study area while the districts that are not touched by this road, including Xaysathan, Thongmixai and Boten, are regarded as minor districts.

**Fig 2:**
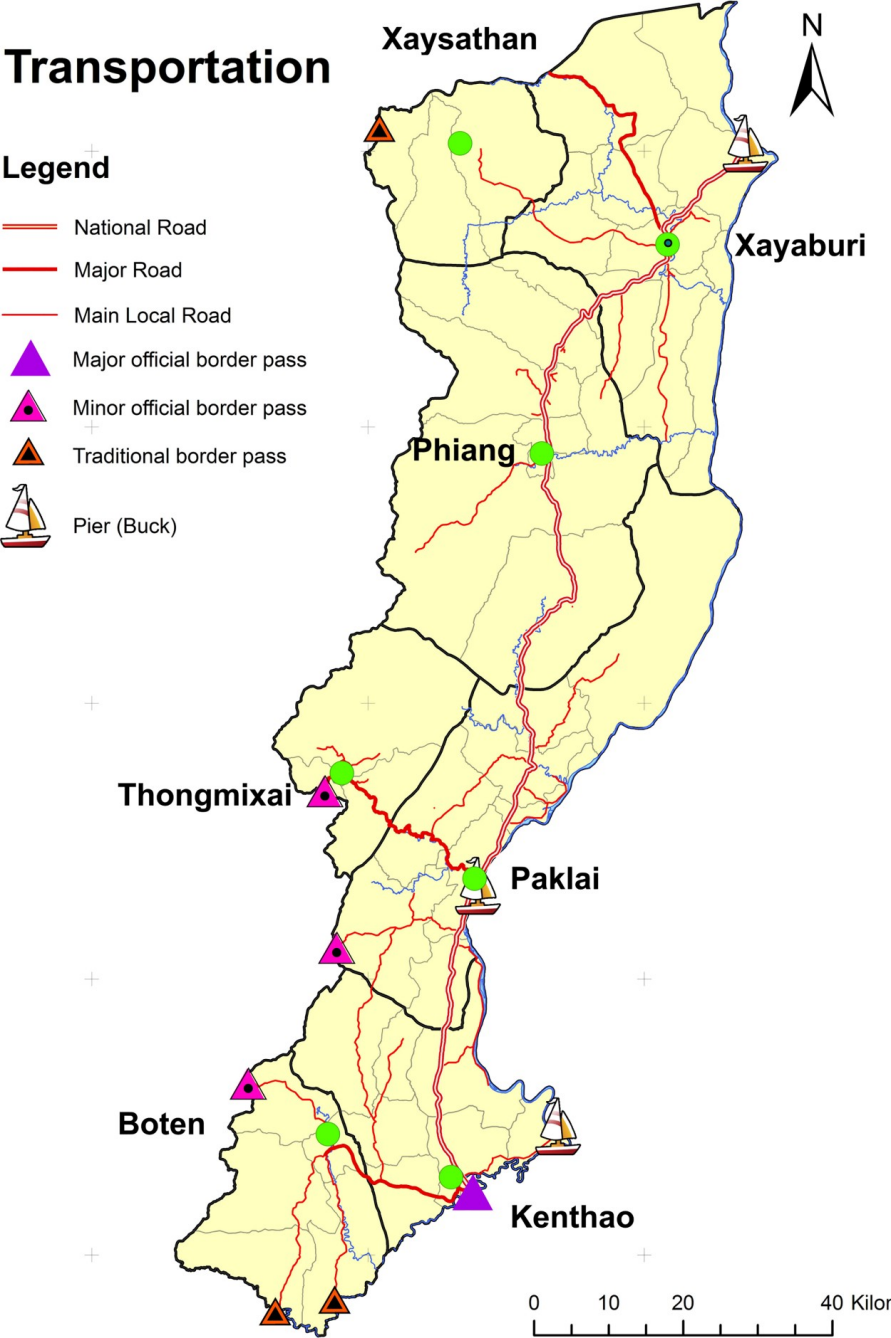
Locations of district centers, main roads and border crossings in the study area. The figure is based on public domain data from Openstreet map and was produced on QGIS 2.16.

Xayaburi town, the provincial capital and the biggest town in the study area, is located in the northernmost part and is connected by road to Luang Prabang City in the north. Urban centers in the south–Paklai town, Kentao town and Meung Mor town—however, are better connected to Thailand which, due to its more highly developed economy, has a strong influence on the economy of Laos as both a source of consumer goods (household utensils, electric appliances, furniture, food ingredients, clothes, etc.) and of demand (for agricultural products such as maize, job’s tear, sesame, peanut and tangerine, etc.). Several border crossings to Thailand are located in the south including the major crossing near Kentao town at the southernmost point of the study area which is the major communication and transportation channel of goods between Thailand and Laos. Therefore, the flow of agricultural products from the study area is mainly directed southwards to Thailand. Fig 2 shows the locations of district centers, main roads and border crossings in the study area.

Market accessibility in the study area decreases along a gradient from south to north. Table 1 shows the evidence from various indicators of market accessibility. Data on market accessibility shown in Table 1 is mainly derived from researcher’s survey conducted in 2011–2012 and from government records.

Southern districts have better access to agricultural markets than districts in the north. As Thailand is the major demand source for agricultural products from Xayaburi, the proximity to major border crossings and the better road conditions in the south as well as the prevalence of level terrain contribute to lower transportation costs. Road conditions are generally better in the south and most villages can be reached by ordinary cars (cars without 4-wheel drive). The lower transportation cost results in a higher selling price for maize, the most popular cash crop in Xayaburi.

Agricultural products are collected and exported by trading companies in Laos. The higher number of trading companies and the higher value of export crop products in the southern districts thus reflects the higher level of agricultural market accessibility. Residents in the south are also more integrated into agricultural markets as can be seen from the higher percentage of cash crop farmers and the higher average income from cash crops per household. In addition, there are more marketplaces in the south, and these are more evenly distributed where most sub-districts have a marketplace, than in the north, where marketplaces are located only along the main road.

### Expansion of cash crop cultivation

The government supports agricultural commercialization in various forms such as opening border crossings, infrastructure development, and land use policies. Government policies on land allocation and relocation are aimed at preventing farmers from growing upland rice in shifting cultivation systems by moving them far away from their traditional upland farm areas and collecting a high tax for this practice. Government support along with the increasing demand for agricultural products from Thailand has led to intensified cross-border trade with Thailand and a huge expansion in agricultural areas since the year 2000. The total agricultural area in the study area increased by 300 km^2^, mostly at the cost of unstocked forests and vegetation regrowth within less than a decade (from 2000 to 2008) according to MAF records and the study of land cover dynamics through the satellite image analysis (see S3 File for summary results of land use change from satellite image analysis and document analysis). The total agricultural area of the study area in 2008 was around 983 km^2^ or 8.8% of the total land area. Agricultural expansion started in the south in the early 2000s while agricultural areas in the north expanded after 2006.

While agricultural area has increased, the share of traditional food crops such as rice (including paddy and upland rice fields) and market cash crops (maize, job’s tear, sesame, peanut, cotton, rubber and cassava) has fluctuated. Although paddy rice can be both a traditional staple and a modern cash crop, this study is limited to regarding rice as a food crop. According to MAF records, the cash crop area in Xayaburi province rose from 9% of the total agricultural area in 1976 to 18% in 2002, and then jumped to 35% in 2005. There was a further jump to 60% in 2010 when maize and other cash crops spread into all districts, eventually surpassing the total rice area. In the 7 districts of the study area, for which data is available since 2006, the cash crop area was 421 km^2^ in 2006 representing 60% of the total agricultural area, then rose to 606 km^2^ (62%) in 2008, and finally to 653 km^2^ (66%) in 2010 (see S4 File for statistics on land use and land use change).

The southern districts are shown in Table 2 to have a greater share of cash crop area of the total agricultural land than the northern districts. In 2008, cash crop areas constituted about 75% of all agricultural land in the major southern districts of Kentao and Paklai, and 40–70% in southern districts located further away from the road. In the major northern districts, on the other hand, the shares of cash crop area to total agricultural land was around 40%. In Xaysathan, the most remote area in the northwestern part of the study area, it was as low as 3%. The share of cash crop area continued to increase and reached 80% in 2010 in the major southern districts and around 60–65% in the minor southern districts. However, it increased only slightly in the north over the same time period.

The survey in 2012 showed that households in the south use on average 80% of their land for cash crop cultivation while households in the north use only 62% of their land for cash crop cultivation (see Table 3). Taking into consideration that household respondents were divided into 3 wealth classes which differ from each other with respect to land possession, the values of standard deviation (provided in brackets in the table) are quite high. Nevertheless, comparing agricultural land use of each class (which has lower value of standard deviation) provides similar differences between the north and the south as those shown in Table 3.

The data from both district and household levels indicates that the proportion of cash crop area to total agricultural area increases with increasing market accessibility.

Increased market accessibility occurs in parallel with increased availability of traders, marketplaces, commodities and information which create better market opportunities for farmers and allow them to start or increase growing cash crops. The major factors behind the decision of farmers to plant maize on a large scale in Xayaburi, according to the household survey, include market certainty, access to credit and inputs, and the desire of farmers to earn more income.

Market certainty for maize results from the high demand for maize in Thailand and the increased availability of maize traders in the study area. Contract farming for maize also provides a guaranteed market for farmers. Through the contract farming system, farmers can get easier access to credit and other inputs for growing maize such as hybrid seeds, pesticide, plowing and fertilizer. While wealthy farmers invest their own money, the household survey found that 53% of maize farmers receive inputs through the contract farming system.

Farmers’ desire for more income is a critical factor in their decision to plant maize rather than upland rice. A comparison between intensive maize cultivation and shifting cultivation with upland rice based on the household survey can help to explain farmers’ decisions. The yield of maize can be expected to be as high as 7 tons / ha and expected farm gate price can be 5 THB / kg. Hence, maize can generate an income about 35,000 THB / ha (1,130 USD approximately in 2011). On the other hand, upland rice produced in shifting cultivation system has an average yield of around 2 tons / ha with the expected farm gate price at 15 THB / kg. In the case that a household keeps a ton of rice for their own consumption, farmers can gain only 15,000 THB / ha (470 USD approximately in 2011). Although the investment in maize for plowing, and for purchasing hybrid seed and pesticides amounts to 9,000 THB / ha on average, which reduces profit to 26,000 THB / ha (840 USD approximately in 2011), the cash income from maize is still higher than that from upland rice. Through the credit for maize contract farming, farmers can get one big cash advance from investors at the beginning of the growing season. It is, therefore, reasonable for farmers to choose planting maize for income over rice for food as they can spend their income from maize on buying rice and other assets as well. Village heads, as key informants, and 58% (38 of 65) of household respondents from the household survey in 8 sample villages in the south mentioned that many farmers in their villages built concrete houses, bought vehicles, and purchased televisions and furniture in the first three years of growing maize. Credit provided to farmers by contract farming constitutes another reason for farmers to grow maize. The success of neighbors who improved their income and overall wellbeing already after the first year of cultivating maize also persuades farmers to adopt maize.

Another supporting factor for increasing the area of cash crop cultivation is the availability of agricultural land. The greater availability of agricultural land in the southern districts, as shown in Table 2, is generally due to the larger amount of level terrain that can be allocated for cultivation. This, in combination with the fact that farmers of the southern districts generally own larger tracts of land as shown in Table 3 can be an explanation why they are potentially able to allocate a larger share of their land for cash crop cultivation. Spearman’s Correlation analysis on data at the household level also confirms larger land possession for southern households and its significant correlation with cash crop cultivation at the value of 0.952 with 0.01 significant level.

Maize accounted for 57% of the total agriculture area followed by paddy rice and upland rice at 23% and 10% respectively in 2008. Excluding rice which we consider as a food crop, maize dominated the total cash crop area at 85%. There is, however, a significant geographical difference. In the major northern districts (Xayaburi and Phiang) maize was planted over a smaller area than rice. It accounted for only around 30% of the total agricultural area but had a high share, at 80%, of the total cash crop area. On the other hand, maize was extremely dominant in major southern districts (Paklai and Kentao) at around 75% of the total agricultural area and 96% of total cash crop area.

Nevertheless maize production has slightly diminished after 2008. In 2011, the share of maize was down to 52% of the total agricultural area or 75% of the total cash crop area. Job’s tear, on the other hand, became the second most important cash crop of the region with 8% of the total agricultural area. In the major northern districts, the area under maize was down to 20–30% of the total agricultural area while Job’s tear rose to 10–20% of the total agricultural area. Meanwhile, in major southern districts, maize was down to around 70% of total agricultural area. On the other hand, Job’s tear and cassava had each increased in share to around 5% of the total agricultural area or 7% of total cash crop area. The increase in Job’s tear and cassava area means that both crops have taken over some of the share from maize. This shift was analyzed through key informant interviews and the household survey. The major push factor for this shift is a set of problems that affected the maize trade. The low selling price of maize in the north, due to limited access to the maize market, greatly affected northern farmers especially when prices fells in 2008. After that, many farmers in the north did not continue maize farming and attempted to find alternative crops. The expansion of the Job’s tear market, due to increasing demand from China and the establishment of processing factories in some major districts, are the pull factors that turned Job’s tear into an alternative for farmers.

### Comparison of income and rice production along a gradient of market accessibility

The expansion of cash crop cultivation resulting from improved market accessibility in the south has strong effects on basic wellbeing. Tables 4 and 5 show that average income from cash crops and average total income are much higher in the southern districts as compared to the north (unit of income for Table 4 is per person and for Table 5 is per household). The data from our household survey in Table 5 show that average income from cash crops is more than twice as high in the south than in the north. Although northern households earn higher non-agricultural income than southern households, this type of income is much less than cash crop income of southern households, resulting in the lower total income for northern households.

The correlation analysis of both village and household level data provides further evidence of the contribution of cash crop cultivation to income. The Pearson’s Correlation Analysis on village level, shown in Table 6, indicates the strong relationship between percentage of cash crop area in total land area and agricultural income as well as total income. The Spearman’s Correlation Analysis on household level, shown in Table 7, also indicates that the larger a household’s cash crop area, the higher its income as well as its wealth (the status of household wealth was quantified into 5 marks from 1 [lowest wealth] to 5[highest wealth] based on village authorities and researcher’s observation).

**Table 6:** The correlation between cash crop area and income and rice production at the village level.

Variables	Agricultural Income	Total income	Rice production
% Cash crop area (sig.)	0.444 ** 0.000	0.393 ** 0.000	0.116 * 0.043

Note

** Correlation is significant at the 0.01 level

* Correlation is significant at the 0.05 level

**Table 7:** The correlation between cash crop area and income and rice production at the household level.

Variables	Agri. income	Total income	Wealth	Rice production
Cash crop area (sig.)	0.746 ** 0.000	0.557 ** 0.000	0.418 ** 0.000	0.289 ** 0.001
% Cash crop area (sig.)	0.549 ** 0.000	0.299 ** 0.001	0.108 0.239	- 0.191 ** 0.036

Note

** Correlation is significant at the 0.01 level

* Correlation is significant at the 0.05 level

(-) Inverse relationship

However, unlike absolute cash crop area, percentage of cash crop area, at the household level, though significantly related to income, is not significantly related to wealth. Therefore, households with a high percentage of cash crop area are not necessarily rich or wealthy, especially those households whose cash crop area is less than 2 ha. On the other hand, households who manage a cash crop area of more than 3 ha have at least a medium level of wealth.

Most of the southern districts, except for Paklai, appear to have higher rice production per person than northern districts despite lower total rice production (see Table 4). The reason for higher rice production of southern farmers is the larger paddy area per household as compared to the north (see Table 2). The survey at household level, nevertheless, produced the contradictory result that northern households produce a slightly higher amount of rice than southern households (see Table 5 [unit for rice production is per household]). Households in both subregions own similar amounts of paddy area but the fact that northern household dedicate a larger amount of land to upland rice cultivation (see Table 3) could be a reason for their altogether higher rice production. The difference between district and household level data can be due to the fact that district data is from 2008 while the household survey was conducted in 2012 when farmers in the south possibly allocated more land for cash crops than for rice.

The Spearman’s Correlation Analysis reveals that households’ total cash crop area significantly correlates with rice production while the share of cash crop area in total land area has an inverse relationship with rice production (see Table 7). On the other hand, the Pearson’s Correlation Analysis at village level found only a minor significant relationship between the share of cash crop area in total agricultural village land and rice production (see Table 6). The inverse or moderately positive relationship between the share of cash crop area in total land area and rice production can be explained by the fact that some households with higher share of cash crop area use less area for rice, particularly households with small agricultural land so that they have lower rice production.

In contrast, a relative larger cash crop area does not necessarily imply a relative lower area for rice cultivation for a household. The cross tabulation analysis between cash crop area and rice production based on the data from household survey shows that most households owning a relative larger cash crop area also own a relative larger paddy area and produce a greater amount of rice (see Table 8). The cross tabulation analysis between agricultural area and rice production based on the data from household survey shows a similar trend (see Table 9). In other words, farmers tend to use part of their land for rice and the rest for cash crop cultivation. Therefore, it can be concluded that whether farmers use part of their land for rice and part for cash crops depends on the size of their landholding. Farmers with small landholdings of less than 2 ha tend to grow rice on most of their agricultural land.

**Table 8:** Cross tabulation analysis between cash crop area and rice production at the household level.

Cash crop area (ha)	Rice production (tons / year)					
	< 1.0	1.0–1.9	2.0–2.9	3.0–3.9	> 4.0	Total
< 1.0	12	13	1	3	6	**35**
1.0–1.9	2	6	8	0	6	**22**
2.0–2.9	3	3	8	3	2	**19**
3.0–3.9	3	7	3	2	3	**18**
4.0–4.9	2	2	1	3	3	**11**
> 5.0	1	2	1	0	12	**16**
**Total**	**23**	**33**	**22**	**11**	**32**	**121**

**Table 9:** Cross tabulation analysis between agricultural area and rice production at the household level.

Agricultural area (ha)	Rice production (tons / year)					
	< 1.0	1.0–1.9	2.0–2.9	3.0–3.9	> 4.0	Total
< 1.0	7	5	0	0	1	**13**
1.0–1.9	6	10	2	3	5	**26**
2.0–2.9	4	7	9	1	3	**24**
3.0–3.9	3	2	3	3	5	**16**
4.0–4.9	2	6	4	2	1	**15**
> 5.0	1	3	4	2	17	**27**
**Total**	**23**	**33**	**22**	**11**	**32**	**121**

However, Table 9 shows that several households do not follow this trend. Thirty percent of households (13 of 43), the majority of them in the north (10 of 13), produce over 3 tons of rice per year but have less than 3 ha of total agricultural land or, in other words, their rice production is higher on a relatively smaller amount of land. On the other hand, 29% of households (12 of 42), the majority of them in the south (8 of 12), own over 4 ha of total agricultural land but produce less than 2 tons of rice per year, i.e. their rice production is lower on a larger land area.

Northern households with, on average, a smaller amount of agricultural land available to them, use a larger amount of their land holding for rice and the remaining land for cash crops. On the other hand, southern households that own larger land areas can use part of the land for rice and still have more land left over than a household in the north to use for cash crops. Adding better level of market accessibility, southern households can earn a much higher income from cash crops due to both higher production and higher selling price than northern households and can still keep a good level of food security. In some cases, households in the south focus on cash crops only and spend their income to buy food. The results thus indicate that in general households in the north focus on rice production while households in the south focus on generating income from cash crops.

Regarding rice sufficiency, the household survey has revealed that most farmers keep rice which they produce primarily for their own consumption and sell the surplus for income only when they can fully satisfy their domestic consumption needs. Rice sufficiency, in this study, can therefore be equated with rice production per household. The results thus indicate that households in southern districts maintain a sufficient level of rice production as well as earn more income from cash crops to buy food, contributing to their basic wellbeing, while households in northern districts earn a much lower income than households in the south, resulting in a lower level of their basic wellbeing.

Government data of poverty incidence, determined by percentage of poor villages and households, reveal a similar trend of wellbeing in the study area. The Government’s standard of poor households is determined through semi-quantitative indicators as not having enough food, adequate clothing, permanent housing or access to health, education or transportation services. Government data show that poverty incidence is more severe in northern districts (see Table 4).

In general, the southern districts of Xayaburi, characterized by better market accessibility, availability of more arable land and more land under cash crop cultivation, have a higher level of basic wellbeing than the northern districts as indicated by higher relative income, lower poverty and higher rice production. This suggests that local people in the south can achieve both income generation and rice sufficiency to improve their basic wellbeing. It also suggests that the ability of households to earn a good income through cash crop cultivation is not necessarily at the cost of rice sufficiency. Spearman’s correlation analysis at the household level confirms the strong positive relationship between income and rice production.

## Discussion

The higher level of market accessibility in the southern part of Xayaburi is due to many factors. Cash crop production in Laos is heavily influenced by the demand of neighboring countries like Thailand, China, and Vietnam [13,16,18]. Southern Xayaburi has advantages of market accessibility due to its proximity to Thailand. The great number of important border crossings facilitates market access across the border. The relatively high selling price of maize in the south in combination with the lowest transportation cost at the shortest distance to market (demand source) leads to the best selling price for local produce as argued by Petron et al [53] and Rodrigue [54]. Bonnin [55] identified four major factors for greater market prevalence and accessibility which includes cross-border trade, physical terrain, road development, and consumer groups. All of these factors are prominent in southern Xayaburi. Access to markets across the border, better road conditions, and a larger amount of level terrain support a higher amount of products, services and traders, and more money value in the southern districts than in the north. All of these factors lead to better market infrastructure and more market availability in the forms of traders, marketplaces, commodities, and information; altogether resulting in a higher market accessibility in the south.

Agrarian change as we can observe in the study area consists of the transformation of subsistence agriculture to commercial agriculture with a strong focus on crop monocultures, especially maize. The main driver of this transformation, together with improving access to agricultural markets, is the demand across the border in the markets of Thailand for maize and in markets of China for other crops such as job’s tear and cassava. This resonates with the observation by Akram-Lodhi and Kay [20] already mentioned above concerning the emphasis on the promotion of an agricultural export-led strategy as the principal means of enhancing rural accumulation. In order to serve these cross-boundary markets, the Lao government supports export-oriented agricultural commercialization by opening border crossings, infrastructure development, and land use policies. Similar developments can be observed within the Mekong region in SW China, where the government promotes rubber monocultures as a means of eradicating poverty [56].

The main intrinsic driving factor of agrarian change as manifested in the study area is the desire of farmers for higher incomes as confirmed by the results of our research. The results show that many households built better houses and bought assets such as vehicles or television sets within 3 years after they started growing maize. Broegaard et al [57] found similar evidence in Huaphan, northern Laos, that farmers spent their income from maize cultivation on household assets or invested in their children’s education. Contract farming provides market opportunities and other incentives for farmers to focus on cash crop cultivation. Improved market access leads to the transformation from a subsistence economy to a market-oriented economy, especially by intensifying cash crop cultivation. Evidence for this transformation in the southern districts includes the higher absolute cash crop area and higher share of cash crop area out of the total agricultural area as well as by the higher percentage of cash crop farmers. Messerli et al [51] reviewed a large number of studies from Laos and found a trend of increasing area of paddy and permanent farmland in areas of high accessibility. The increasing portion of agricultural land under cash crop cultivation shows that farmers alter their farming practices to adapt to emerging market opportunities, as argued by a number of studies [17,18,58,59]. As asserted by Mertz et al [60], market opportunities following infrastructure improvement can act as pull factors for farmers to change from traditional upland rice to permanent agriculture. This has been observed in many areas of Southeast Asia [10] as well as in India and Africa [35]. The difference between households in the northern and southern parts of Xayaburi with respect to the share of cash crop area reflects that farmers respond differently to changing market situations depending on their access to markets, knowledge, information, labor, natural resources and social status as argued by Douxchamps et al [8], Ellis [44] and Wiesmann [52].

Cash crop cultivation allows farmers to earn higher incomes especially in southern Xayaburi, while maintaining a level of rice sufficiency that is still much higher than the Lao National rice security standard. Income from cash crops constitutes a higher share than incomes from other sources in the total income of southern farmers. The Government’s goal of improving living standards through market accessibility and cash crop cultivation [6,14,32,61] thus is achieved in southern Xayaburi. Through the combination of the two, farmers in the south experience higher standards of basic wellbeing than farmers in the north who rely more strongly on subsistence agriculture. Similar to the case of northern Xayaburi, farmers in areas with low market accessibility in Romania [5] and India [19] produced more food, but had a low income and a lower level of basic wellbeing.

There is a concern that replacement of food crop production by cash crop cultivation due to improved market accessibility may result in food insecurity when market failures reduce incomes and the ability to buy food. This has been proven to constitute a serious concern through many case studies in developing countries such as Guatemala [38], Tanzania [47], Nigeria and South Africa [30], Mozambique [46], the Meghalaya plateau in India [27] and also in Laos [62]. Broegaard et al [57] found that the shift towards maize cultivation in Laos created pressure on land and diminished the share of wild food in daily diets and thus the quality of nutrition. With respect to staple crops, the situation is different in Xayaburi, where cash crop cultivation has not replaced rice production. Households in the south of Xayaburi grow just enough rice for household-consumption, which is typically around 1–2 ha, and devote the rest of their land to cash crops. The strategy to keep a part of land for rice to meet household minimum demand is a key to alleviating the risks of rice insufficiency that may result from cash crop failure and market uncertainties. Rice insufficiency, in the sense of not enough rice being available for household consumption, can become a problem of farmers who grow only a cash crop when the crop is damage by disaster or when the price of the crop goes down, so that farmers do not have enough money to buy rice. This strategy fits the multi-faceted strategies proposed by Wiesman [52] and matches suggestions from Immink and Alarcon [38], Hazra [28] and Porter and Howard [30] of balancing food crops and cash crops by increasing productivity for food crops and lowering the risks from cash crop production and marketing. The same strategy was also observed in many other areas in Southeast Asia [10].

The strategy of combining cash cropping with a minimum of food production to meet domestic demand is more effective when the household owns a sufficiently large amount of land as is the case in southern Xayaburi. Higher availability of agricultural land thus is a critical factor for supporting farmers in capitalizing on market opportunities and expanding cash crop cultivation. In the same way, some studies [19,63] argue that small land size is a major limitation preventing poor farmers from crop diversification or achieving income generation from cash crop cultivation. To reduce the limitations imposed by the availability of land, Douxchamp et al [8] and Mabiso et al [46] suggest intensification as a means for increasing productivity. The correlation analysis showing that larger areas of cash crops strongly correlate with good basic wellbeing confirms the importance of land availability. The correlation analysis also shows that larger area of cash crops is a stronger factor than higher rice production in influencing basic wellbeing positively. The greater amount of land suitable for cultivation in the south is to some extent due to a larger proportion of level terrain, as compared to the north. Geographical conditions thus provide farmers in southern Xayaburi with an advantage that contributes to the improvement of their basic wellbeing.

Another major concern in the context of agrarian transformation to commercial cash cropping is vulnerability to market inadequacies and uncertainties, in other words, that cash crop specialization may increase the danger of income loss from crop failure, price fluctuation, inefficient market institutions and exploitation from buyers [10,30,38,44]. Income loss and decreased food production caused by the agrarian transformation can result in food insecurity and lower level of wellbeing [29,46]. Many studies found that reduction of income from cash crop cultivation is mainly due to the lack of well-functioning markets and to low market accessibility [17,27,47]. This is the case in the north of Xayaburi, where accessibility to maize markets is relatively lower than in the south resulting in lower selling price and lower incomes of farmers which make them susceptible to price fluctuations. For this reason, many northern farmers have abandoned maize and turned to other crops or back to upland rice after the drop in prices for maize in 2008. The same drop of price for maize in 2008 caused many farmers in Huaphan, northeastern Laos, to change their crop from maize back to upland rice [64]. Similarly the drop of rubber prices in 2011 caused many farmers in Luang Namtha, northern Lao and Xishuangbanna, in the southern part of Yunnan Province, China [65] to change their cash crop to banana.

Although price fluctuations are a feature also in the southern districts of Xayaburi, farmers there are still able to make a profit as the selling price is comparatively higher than in the north due to better market access. This reflects that the vulnerability of farmers engaging in cash crop agriculture can be mitigated through good market accessibility, in other words, market accessibility is another key to achieve cash crop cultivation and improved basic wellbeing of farmers [8,10,27,47]. Messerli et al [51] confirm that accessibility is the key to poverty alleviation and to better rural wellbeing. Though improved market accessibility may lead to lower rice production, the impact is not so significant when farmers still grow rice to meet their minimum consumption needs, and when the income from cash crops, enhanced by improved market accessibility, can compensate for losses from rice production. Market accessibility can be improved not only by infrastructure improvement but also through selection of crops that have marketing potential in a specific area. For instance, farmers in the northern districts of Xayaburi have recently started to grow Job’s tear in response to demand by Chinese traders and the establishment of Chinese Job’s tear factories. In this way, Job’s tear has emerged as an alternative crop to maize. Job’s tear also requires less investment than maize, thus allowing farmers easier access to Job’s tear commercial markets. The replacement of rubber by banana in Xishuanbanna and Luang Namtha [65] also suggests the contribution of crop selection strategy to farmer’s market accessibility.

The case of Xayaburi has demonstrated that three factors: availability of land, good market accessibility and the strategy to keep a part of land for rice to meet household minimum demand, are instrumental for farmers in southern Xayaburi to achieve higher income through market-based agriculture without facing the problem of rice insufficiency. These three key factors are interrelated and can support one another or compensate for one another when one of them faces limitations. Larger amount of agricultural land makes it easier for farmers to respond to market opportunities by increasing the cash crop component in their farming strategy while accessibility improvement increases these opportunities. The strategy to keep a portion of land for rice to meet a household’s minimum demand for rice, lessens the risks of rice insufficiency. This finding matches the argument by Douxchamps et al [8] and Turner et al [17] that wellbeing is determined by market accessibility, land possession and economic diversification.

Among the three factors, the strategy of farmers to keep a portion of land for rice to maintain rice sufficiency is probably the most critical. The case of Huaphan in northeastern Lao where maize was grown in monocultures shows that though farmers were able to raise their income and improve their basic wellbeing initially, they faced the problem of profit loss and rice shortage when the price of maize came down [64]. In the case of Xishuanbanna and Luang Namtha [65,66] where rubber has been monocropped, farmers faced similar problems due to a drop of rubber prices in 2011. Therefore farmers with good market access and sufficient agricultural land can still face the issue of income loss or rice insufficiency if they pursue the strategy of cash crop monoculture. The reasons why farmers in Xayaburi keep part of their land for rice are difficult to ascertain and require more research. They may include that rice can be both food and cash crop, but also that rice can be grown in different environments even in the steep hill or mountain areas that are less suitable for certain cash crops [67]. The cultural values of rice can also play a role, especially in Laos, where rice is consumed not only because of its nutritional but also because of its spiritual value [68].

## Conclusion

The southern part of Xayaburi Province has a higher level of market accessibility than the north due to several factors, including better transportation infrastructure, greater amount of level terrain, more border crossings, and greater proximity to demand sources. The northern part of Xayaburi Province, despite the fact that the provincial capital is located there, cannot provide the same level of market functions mainly because of its distance from demand sources. Greater or improved market accessibility especially in the south has led to the expansion of cash crop cultivation and the shift to market-based agriculture. Cash crops particularly maize have become popular and to some extent have replaced rice, the traditional food crop.

In general, we found that farmers in the southern districts of Xayaburi were able to improve their basic wellbeing through generating more income while retaining their ability to secure rice production above the country’s standard line. On the other hand, farmers in the northern districts of Xayaburi, where market accessibility is lower and availability of arable land is more limited, generally maintain a higher level of subsistence agriculture, but cannot generate enough profit through cash crop cultivation to considerably improve their basic wellbeing. Some of them lose money due to fluctuations of the maize price while others face problems of rice insufficiency regardless of whether they practice more traditional subsistence production or intensified cash crop cultivation.

We identified three key factors for farmers in the south which are keys to overcoming the concerns of rice insufficiency and market uncertainties and to improving their basic wellbeing: availability of land, good market accessibility and the strategy of retaining a part of their land for rice cultivation in order to meet minimum household consumption needs. As most agriculture dependent countries are undergoing similar pathways of agrarian transformation, these key factors can be applied to a wider geographical area to improve farmers’ basic wellbeing. Governments and farmers can improve these factors through various means or strategies. Governments can improve market accessibility through investment in market infrastructure development while farmers can enhance their market access through crop selection strategies that fit the market potential of the area. Governments can enhance availability of agricultural land through change in land allocation policy while farmers can enhance productivity of their land through intensification strategies. Governments should encourage or provide incentives for farmers to produce enough food or to maintain this strategy where it is already in place such as in southern Xayaburi. Those key factors should be encouraged in development policy and planning to achieve wellbeing improvements for rural farmers through market-based agriculture channels.

## Supporting information

S1 File Data from Lao Government. This file contains the table showing the information of data obtained from the government which include the name of the data sets, the exact government unit, obtained format and period for which the data were obtained.
(DOCX)

S2 File Household Interview Form. This file is the household interview form that is translated from the original version written in Laos.
(DOC)

S3 File Summary results of land use change from satellite image analysis and document analysis. This file contains the study of land use change of the study area from satellite image analysis done by Boris Führer (unpublished work for Centre of Development and Environment, University of Bern, 2012) and document analysis from MAF and DAFOs.
(DOCX)

S4 File Supplementary statistic data on land use and land use change. This file contains tables providing statistical data derived from government documents (MAF, Xayaburi PAFO and DAFOs) regarding land use change in Xayaburi province and the study area.
(DOCX)

S5 File Data from household survey. This excel file contains raw data of the household survey regarding personal, agricultural and market data of 121 household samples from 15 different villages in the study area.
(XLS)

S6 File Selection of sample villages for the household survey. The file explains the criteria for selecting sample villages for the household survey. It also contains a table that reveals the different characteristics of sample villages along the criteria and a figure that illustrates the locations of sample villages.
(DOCX)

## References

[1] Chambers R. What is Poverty? Who asks? Who Answer? In: International Poverty Centre. Poverty in Focus: What is Poverty? Concepts and Measures. Brazilia: UNDP (United Nations Development Programme); 2006 pp. 3–4. Available from: www.ipc-undp.org/pub/IPCPovertyInFocus9.pdf.

[2] Edward P. The Ethical Poverty Line: A Moral Definition of Absolute Poverty In: International Poverty Centre. Poverty in Focus: What is Poverty? Concepts and Measures. Brazilia: UNDP (United Nations Development Programme); 2006 pp. 14–16. Available from: www.ipc-undp.org/pub/IPCPovertyInFocus9.pdf.

[3] Kakwani N. Poverty and Wellbeing In: International Poverty Centre. Poverty in Focus: What is Poverty? Concepts and Measures. Brazilia: UNDP (United Nations Development Programme); 2006 pp. 20–21. Available from: www.ipc-undp.org/pub/IPCPovertyInFocus9.pdf.

[4] IFAD (International Fund for Agricultural Development). Rural Poverty Report 2011 Rome, Italy. Rome: IFAD; 2010. Available from: www.ifad.org/rpr2011/index.htm.

[5] Alexandri C, Luca L, Kevorchian C. Subsistence Economy and Food Security–the Case of Rural Households from Romania. Procedia Economics and Finance 2015; 22: 672–680.

[6] Barrios E B. Infrastructure and Rural Development: Household Perception on Rural Development. Progress in Planning. 2008; 70: 1–44.

[7] Anriquez G, Stamoulis K. Rural Development and Poverty Reduction: Is Agriculture Still the Key?; 2007. ESA (Agricultural Development Economics Division, FAO) Working Paper No. 07–02. Available from: www.fao.org/3/a-ah885e.pd.

[8] Douxchamps S, Van Wijk M T, Silvestri S, Moussa A S, Quiros C, Ndour N, et al Linking Agricultural Adaptation Strategies, Food Security and Vulnerability: Evidence from West Africa. Regional Environmental Change. 2016; 16(5): 1305–1317. 10.1007/s10113-015-0838-6

[9] FAO (Food and Agriculture Organization of the United Nation). Reducing Poverty and Hunger: the Critical Role of Financing for Food, Agriculture and Rural Development; 2002. Paper prepared for the International Conference on Financing for Development. 18–22 March 2002, Monterrey, Mexico. Available from: www.fao.org/docrep/003/Y6265e/Y6265e00.htm.

[10] Schreinemachers P, Frohlich H L, Clemens G, Stahr K. 2013 From Challenges to Sustainable Solutions for Upland Agriculture in Southeast Asia In: Sustainable Land Use and Rural Development in Southeast Asia: Innovations and Policies for Mountainous Areas. Stuttgart: Springer; 2013. pp. 3–27. Available from: link.springer.com/ chapter/10.1007%2F978-3-642-33377-4_1.

[11] Scott J C. The Art of Not Being Governed: An Anarchist History of Upland Southeast Asia. New Haven: Yale University Press; 2009 Available from: www.law.yale.edu/system/files/documents/pdf/…/LTW-Scott.pdf.

[12] WFP (World Food Programme). Market Analysis Tool: Market Integration. 2007. In: WFP [Internet]. Available from: www.wfp.org/content/market-analysis-tool-market-integration.

[13] Andersson M, Engvall A, Kokko A. Regional Development in Lao PDR: Growth Patterns and Market Integration; 2007. Stockholm School of Economics Working Paper 234. Available from: http://swopec.hhs.se/eijswp/papers/eijswp0234.pdf.

[14] Rigg J D. Forests, Marketization, Livelihoods and the Poor in the Lao PDR. Land Degradation and Development. 2006; 17 (2): 123–133. 10.1002/ldr.719

[15] Lambin E F, Tuner B L, Geist H J, Agbola S B, Angalsen A, Bruce J W, et al The Causes of Land-use and Land-cover Change: Moving Beyond the Myths. Global Environmental Change. 2001; 11: 261–269.

[16] Rigg J D. Living with Transition in Laos–Market Integration in Southeast Asia. New York: Routledge; 2005 10.4324/9780203002032

[17] Turner B L, Hyden G, Kates R W. Population Growth and Agricultural Change in Africa. Gainsville: University Press of Florida; 1993.

[18] WFP (World Food Programme). Agriculture in Transition: The Impact of Agricultural Commercialization on Livelihoods and Food Access in Lao PDR. Rome: WFP; 2009 Available from: http://rightslinklao.org/wp-content/uploads/downloads/2014/11/WFP-Agric_Transition_Laos.pdf.

[19] Patel K, Gataula H, Johnson D, Karthikeyan M. The Interplay between Household Food Security and Wellbeing among Small-Scale Farmers in the Context of Rapid Agrarian Change in India. Agriculture & Food Security. 2015; 4 (10): 1–16. 10.1007/s10460-016-9740-1

[20] Akram-Lodhi H, Kay C. Neoliberal Globalisation, the Traits of Rural Accumulation and Rural Politics: the Agrarian Change Question in the Twentieth Century In: Peasants and Globalization: Political Economy, Rural Transformation and the Agrarian Question. 2008 pp: 315–338. London: Routledge.

[21] Castella J C. Agrarian Transition and Farming System Dynamics in the Uplands of South-East Asia; 2012. The 3rd International Conference on Conservation Agriculture in Southeast Asia. Hanoi, Vietnam. Available from: http://horizon.documentation.ird.fr/exl-doc/pleins_textes/divers13-06/010058352.pdf.

[22] Rigg J D, Salamanca A, Parnwell M. Joining the Dots of Agrarian Change in Asia: A 25 Year View from Thailand. World Development. 2012; 40 (7): 1469–1481.

[23] Thongmanivong S, Phanvilay K, Fujita Y, Fox J. Agrarian Land-Use Transformation in Northern Laos; 2006. Sustainable Sloping Lands and Watershed Management Conference. December 12–15, 2006. Luang Prabang, Lao PDR. Available from: http://rightslinklao.org/wp-content/uploads/downloads/2014/11/ch2_07_sithong.pdf.

[24] Zorya S, Mahdi S. High Marketing Costs and Inefficient Policies in Tanzania’s Maize Market: A Poverty Perspective; 2009 Draft Note. Available from: www.tzdpg.or.tz/fileadmin/_migrated/content_uploads/The_Maize_Market_Note_-WB_SZ.pdf.

[25] Zeller M, Diagne A, Mataya C. Market Access by Smallholder Farmers in Malawi: Implications for Technology Adoption, Agricultural Productivity, and Crop Income; 1997. IFPRI (International Food Policy Research Institute). FCND (Food Consumption and Nutrition Division) Discussion Paper No.35. Available from: http://ageconsearch.umn.edu/bitstream/97054/2/Market%20access%20by%20smallholder%20farmers%20in%20Malawi.pdf.

[26] Hanssen C H. Lao Land Concessions, Development for the People; 2007. International Conference on Poverty Reduction and Forest: Tenure, Market and Policy Reforms. 3–7 September 2007. Bangkok, Thailand. Available from: www.recoftc.org/site/fileadmin/docs/Events/RRI_Conference/Proceedings/Paper_20_Hanssen.pdf.

[27] Bahera R N, Nayak D K, Anderson P, Maren I E. From Jhum to Broom: Agricultural Land-use Change and Food Security Implications on the Meghalaya Plateau, India. Ambio. 2015 Available from: http://link.springer.com/article/10.1007%2Fs13280-015-0691-3. 10.1007/s13280-015-0691-3 PMC4709356 26254789

[28] Hazra C R. Crop Diversification in India In: Crop diversification in the Asia-Pacific Region. Bangkok: FAO (Food and Agriculture Organization of the United Nations); 2001 pp: 32–50. Available from: ftp://ftp.fao.org/docrep/fao/003/x6906e/x6906e00.pdf.

[29] Kahane R, Hodgkin T, Jaenicke H, Hoogendoorn C, Hermann M, Keatinge J D H, et al Agrobiodiversity for Food Security, Health and Income. Agronomy for Sustainable Development. 2013; 33 (4): 671–693. 10.1007/s13593-013-0147-8

[30] Porter G, Howard K P. Comparing Contracts: An Evaluation of Contract Farming Schemes in Africa. World Development. 1997; 25 (2): 227–238. 10.1016/S0305-750X(96)00101-5

[31] Rahman S. Whether Crop Diversification is a Desired Strategy for Agricultural Growth in Bangladesh? Food Policy. 2009; 34: 340–349. 10.1016/j.foodpol.2009.02.004

[32] Bank World. Lao People’s Democratic Republic: Policy, Market, and Agriculture Transition in the Northern Uplands. New York: The World Bank; 2008 Available from: http://laocs-kis.org/wp-content/uploads/2015/02/10.-Policy-Market-and-Agriculture-transition-2008.pdf.

[33] Zimemrer K S, Carney J A, Vanek S J. Sustainable Smallholder Intensification in Global Change? Pivotal Spatial Interactions, Gendered Livelihoods, and Agrobiodiversity. Current Opinion in Environmental Sustainability. 2015; 14: 49–60. 10.1016/j.cosust.2015.03.004

[34] Bijman J. Contract Farming in Developing Countries: An Overview; 2008. Wageningen University and the Netherlands Ministry of Foreign Affairs (DGIS) Working Paper. Available from: www.wur.nl/upload_mm/5/c/b/79333121-6f4b-4f86-9e8e-0a1782e784d6_ReviewContractFarming.pdf.

[35] Carswell G. Agricultural Intensification and Rural Sustainable Livelihoods: A ‘Think Piece’; 1997. IDS (Institute of Development Studies) Working Paper 64. Available from: https://www.ids.ac.uk/files/dmfile/Wp64.pdf.

[36] Tipraqsa P, Schrienemachers P. Agricultural Commercialization of Karen Hill Tribes in Northern Thailand. Agricultural Economy. 2009; 40 (1): 43–53. doi: 10.1111/ j.1574-0862.2008.00343.x

[37] Eder J F. Agricultural Intensification and Labor Productivity in a Philippine Vegetable Gardening Community: a Longitudinal Study. Human Organization. 1991; 50 (3): 245–55. 10.17730/humo.50.3.h65j4523w6114380

[38] Immink M D C, Alarcon J A. Household Food Security and Crop Diversification among Smallholder Farmers in Guatemala–Can Maize and Beans Save the Days?; 1991 FAO (Food and Agriculture Organization of the United Nations) Available from: www.fao.org/docrep/u8050t/u805t06.htm.

[39] Borras S M. Jr Agrarian Change and Peasant Studies: Change, Continuity and Challenges–an Introduction. The Journal of Peasant Studies. 2009; 36 (1): 5–31. 10.1080/03066150902820297

[40] Powell B, Thilsted S, Ickowitz A, Termote C, Sunderland T, Herforth A. Improving Diets with Wild and Cultivated Biodiversity from Across the Landscape. Food Security. 2015; 7 (3): 535–554. 10.1007/s12571-015-0466-5

[41] Krahn J. Cooking Up: Dietary Change in Lao Upland Kitchens. Juth Pakai. 2003; 1: 4–15.

[42] WFP (World Food Programme). Lao PDR: Comprehensive Food Security and Vulnerability Analysis (CFSVA). Vientiane: WFP; 2007. Available from: documents.wfp.org/stellent/groups/public/documents/ena/wfp178397.pdf

[43] Wright S. Agriculture in Transition: The Impact of Agricultural Commercialization on Livelihoods and Food Access in the Lao PDR. Vientiane: WFP; 2009 Available from: www.directoryofngos.org/ingo2/a/download?id=document1958&field=file&notetype=document&file=Ny5fQWdyaWN1bHR1cmVfaW5fVHJhbnNpdGlvbl8yMDA5LnBkZg==

[44] Ellis F. Peasant Economics: Farm Households and Agrarian Development. Cambridge: Cambridge University Press; 1988.

[45] Pultrone C. An Overview of Contract Farming: Legal Issues and Challenges In: Uniform Law Review. Rome: FAO (Food and Agriculture Organization of the United Nations), 2012 pp: 262–289. Available from: www.fao.org/fileadmin/user_upload/contract_farming/Uniform%20Law%20review_Pultrone.pdf.

[46] Mabiso A, Cunguara B, Benfica R. Food (In)security and its Drivers: Insights from Trends and Opportunities in Rural Mozambique. Food Security. 2014; 6(5): 649–670. 10.1007/s12571-014-0381-1

[47] Haug R, Hella J. The Art of Balancing Food Security: Securing Availability and Affordability of Food in Tanzania. Food Security. 2013; 5(3): 415–426. 10.1007/s12571-013-0266-8

[48] Fukuda-Parr S. The Human Poverty Index: A Multidimensional Measure In: International Poverty Centre. Poverty in Focus: What is Poverty? Concepts and Measures. Brazilia: UNDP (United Nations Development Programme); 2006 pp. 7–9. Available from: www.ipc-undp.org/pub/IPCPovertyInFocus9.pdf.

[49] Laderchi C, Saith R, Stewart F. 2006 Does the Definition of Poverty Matter? Comparing Four Approaches In: International Poverty Centre. Poverty in Focus: What is Poverty? Concepts and Measures. Brazilia: UNDP (United Nations Development Programme); 2006. pp. 10–11. Available from: www.ipc-undp.org/pub/IPCPovertyInFocus9.pdf.

[50] Heinimann A. Pattern of Land Cover Change in the Lower Mekong Basin, the Relevance of Mesoscale Approaches. PhD Thesis, Centre for Development and Environment (CDE), University of Bern. 2006. Available from: https://cdeweb4.unibe.ch/Pages/Publication/1072/Pattern-of-Land-Cover-Change-in-the-Lower-Mekong-Basin-The-role-of-mesoscale-approaches.aspx.

[51] Messerli P, Bader C, Hett C, Epprecht M, Heinimann A. Towards a Special Understanding of Trade-Offs in Sustainable Development: A Meso-Scale Analysis of the Nexus between Land-Use, Poverty, and Environment in the Lao PDR. PLoS ONE. 2015; 10 (7): e0133418 10.1371/journal.pone.0133418 26218646 PMC4517898

[52] Wiesmann U. Sustainable Regional Development in Rural Africa: Conceptual Framework and Case Studies from Kenya. Bern: Institute of Geography, University of Bern; 1998.

[53] Pelton L E, Strutton D, Lumpkin J R. Marketing Channels–A Relationship Management Approach. New York: McGraw-Hill Inc; 2002.

[54] Rodrigue J P. The Geography of Transport Systems. 3rd ed New York: Routledge; 2013.

[55] Bonnin C. Markets in the Mountains: Upland Trade-Scape, Trader Livelihoods, and State Development Agendas in Northern Vietnam. PhD Thesis, Department of Geography, McGill University. 2011. Available from: http://digitool.library.mcgill.ca/R/?func=dbin-jump-full&object_id=107672&local_base=GEN01-MCG02.

[56] Ahrend A, Hollingsworth P M, Ziegler A D, Fox J F, Chen H, Su Y, et al Current trends of rubber plantation expansion may threaten biodiversity and livelihoods. Global Environmental Change. 2015; 34: 48–58. 10.1016/j.gloenvcha.2015.06.002

[57] Broegaard R B, Rasmussen L V, Dawson N, Mertz O, Vongvisouk T, Gragan G. Wild Food Collection and Nutrition under Commercial Agriculture Expansion in Agriculture-Forest Landscape. Forest Policy and Economics. 2017; 84: 92–101. 10.1016/j.forpol.2016.12.012

[58] Rudel T K, Schneider L, Uriarte M, Turner B L, DeFries R, Lawrence D, et al Agricultural Intensification and Changes in Cultivated Areas, 1970–2005. PNAS. 2009; 106 (49): 20675–20680. 10.1073/pnas.0812540106 19955435 PMC2791618

[59] Thongmanivong S, Fujita Y. Recent Land Use and Livelihood Transitions in Northern Laos. Mountain Research and Development. 2006; 26 (3): 237–244. doi: 10.1659/ 0276-4741(2006)26[237:RLUALT]2.0.CO;2

[60] Mertz O, Wadley R L, Christiensen A E. Local Land Use Strategies in a Globalizing World: Subsistence Farming, Cash Crops and Income Diversification. Agricultural Systems. 2005; 85: 209–215. 10.1016/j.agsy.2005.06.007

[61] GoL (Government of Lao). National Socio-economic Development Plan (2006–2010). Vientiane: Committee for Planning and Investment; 2006.

[62] Castella J C, Jobard E, Lestrelin G, Nanthavong K, Lienhard P. Maize Expansion in Xieng Khouang Province, Laos: What Prospects for Conservation Agriculture?; 2012. International Conference on Conservation Agriculture and Upland Livelihoods: Innovation for, with, and by Farmers to Adopted to Local and Global Changes: Proceeding: 300–304. CIRAD (French Agricultural Research Centre for International Development). Montpellier, France. Available from: www.asia-uplands.org/Catch-Up/pdf/12CA_C3.pdf.

[63] FAO (Food and Agriculture Organization of the United Nation). Towards the Future We Want. End Hunger and Make the Transition to Sustainable Agricultural and Food Systems. Rome: FAO; 2012.

[64] Willi Y. 2011. Of Maize and Men–Contract Farming in the Houaphanh Province of the Lao PDR: Scale, Scope and Impacts on Peasant Livelihoods. M.Sc. Thesis, Centre for Development and Environment (CDE), University of Bern. 2006.

[65] Yi Z F, Cannon C H, Chen J, Ye C X, Swetnam R D. Developing Indicators of Economic Value and Biodiversity Loss for Rubber Plantations in Xishuangbanna, Southwest China: A Case Study from Menglun Township. Ecol Indic. 2014; 36: 788–797. 10.1016/j.ecolind.2013.03.016

[66] Chen H, Yi Z F, Schmidgt-Vogt D, Ahrends A, Beckschäfer P, Kleinn C, et al Pushing the Limits: The Pattern and Dynamics of Rubber Monoculture Expansion in Xishuangbanna, SW China. PLoS ONE. 2016; 11(2): e0150062 10.1371/journal.pone.0150062 26907479 PMC4764337

[67] IRRI (International Rice Research Institute). Rice Knowledge Bank [Internet]. 2013. Available from: www.knowledgebank.irri.org.

[68] Gomez K A. Rice, The Grain of Culture; 2001. Paper for Siam Society Lecture Series 20 September 2011, Bangkok, Thailand. Available from: www.thairice.org/html/article/pdf_files/Rice_thegrain_of_Culture.pdf.

