# Supplementary material for: Balancing cash and food: The impacts of agrarian change on rural land use and wellbeing in Northern Laos
Source: PLoS One. 2018 Dec 31;13(12):e0209166. doi: 10.1371/journal.pone.0209166 (PMC6312269; doi:10.1371/journal.pone.0209166)
Supplement: S1 File — This file contains the table showing the information of data obtained from the government which include the name of the data sets, the exact government unit, obtained format and period for which the data were obtained. (DOCX) [file pone.0209166.s001.docx]

**Data from Lao Government**

| **Data** | **Source** | **Government unit** | **Obtained format** | **Period the data obtained** |
| --- | --- | --- | --- | --- |
| General Geographical Conditions | Annual report of Xayaburi Provincial Agriculture and Forestry Office: Summary of the Year 2008 – 2009 | Xayaburi PAFO | Hardcopy | Mar 2011 |
| Demographic statistics in Xayaburi and Xaysathan 2008 | Statistic year book of Xayaburi district 2008 | Xayaburi DPI | Hardcopy | Mar 2011 |
| Demographic statistics in Phiang 2008 | Statistic records 2008 – 2009 of Phiang district (from Phiang DPI database) | Phiang DPI | Excel file | Mar 2011 |
| Demographic statistics in Paklai 2008 | Annual statistic records of Paklai district 2008 (from Paklai socio-economic database) | Paklai DPI | Hardcopy | Mar 2011 |
| Demographic statistics in Thongmixai 2008 | Statistic records 2008 – 2009 of Thongmixai district (from Thongmixai DPI database) | Thongmixai DPI | Hardcopy | Mar 2011 |
| Demographic statistics in Kentao 2008 | Statistic records 2008 – 2009 of Kentao district (from Kentao DPI database) | Kentao DPI | Excel file | Mar 2011 |
| Demographic statistics in Boten 2008 | Statistic records 2008 – 2009 of Boten district (from Boten DPI database) | Boten DPI | Excel file | Mar 2011 |
| Markets, trading companies and trading context in Xayaburi and Xaysathan 2011 | Annual Report of Xayaburi District Office of Industry and Commerce: Summary of the year 2010 – 2011 and direction for the year 2011 – 2012 | Xayaburi DOIC | Hardcopy | Nov 2011 |
| Markets, trading companies and trading context in Phiang 2011 | Annual Report of Phiang District Office of Industry and Commerce: Summary of the year 2010 – 2011 and direction for the year 2011 – 2012 | Phiang DOIC | Hardcopy | Nov 2011 |
| Markets, trading companies and trading context in Paklai 2011 | Annual Report of Paklai District Office of Industry and Commerce: Summary of the year 2010 – 2011 and direction for the year 2011 – 2012 | Paklai DOIC | Hardcopy | Feb 2012 |

| **Data** | **Source** | **Government unit** | **Obtained format** | **Period the data obtained** |
| --- | --- | --- | --- | --- |
| Markets, trading companies and trading context in Thongmixai 2011 | Annual Report of Thongmixai District Office of Industry and Commerce: Summary of the year 2010 – 2011 and direction for the year 2011 – 2012 | Thongmixai DOIC | Hardcopy | Feb 2012 |
| Markets, trading companies and trading context in Kentao 2011 | Annual Report of Kentao District Office of Industry and Commerce: Summary of the year 2010 – 2011 and direction for the year 2011 – 2012 | Kentao DOIC | Hardcopy | Feb 2012 |
| Markets, trading companies and trading context in Boten 2011 | Annual Report of Boten District Office of Industry and Commerce: Summary of the year 2010 – 2011 and direction for the year 2011 – 2012 | Boten DOIC | Hardcopy | Feb 2012 |
| Government policies related to agricultural commercialization | National Socio-Economic Development Plan (2006 – 2010)  National Socio-Economic Development Plan (2011 – 2015) | Government of Lao  Government of Lao | PDF file  PDF file | Sep 2009  Mar 2011 |
| Agricultural statistics in Xayaburi and Xaysathan 2006, 2008, 2010, 2011 | 5-Year Summary of Xayaburi District Agriculture and Forestry Office: 2006 – 2010  Annual Report of Xayaburi District Agriculture and Forestry Office: 2011 – 2012 | Xayaburi DAFO  Xayaburi DAFO | Hardcopy  Word file | Mar 2011  May 2012 |
| Agricultural statistics in Phiang 2006, 2008, 2010, 2011 | 5-Year Summary of Socio-economic Performance of Phiang District : 2005 – 2009  Annual Report of Phiang District Agriculture and Forestry Office: 2010 – 2011    Annual Report of Phiang District Agriculture and Forestry Office: 2011 – 2012 | Phiang DPI  Phiang DAFO  Phiang DAFO | Hardcopy  Hardcopy  Word file | Mar 2011  Nov 2011  May 2012 |

| **Data** | **Source** | **Government unit** | **Obtained format** | **Period the data obtained** |
| --- | --- | --- | --- | --- |
| Agricultural statistics in Paklai 2006, 2008, 2010, 2011 | 5-Year Summary of Paklai District Agriculture and Forestry Office: 2006 – 2010  Annual Report of Paklai District Agriculture and Forestry Office: 2011 – 2012 | Paklai DAFO  Paklai DAFO | Hardcopy  Hardcopy | Mar 2011  May 2012 |
| Agricultural statistics in Thongmixai 2006, 2008, 2010, 2011 | Annual Report of Thongmixai District Agriculture and Forestry Office: 2006 – 2007  Annual Report of Thongmixai District Agriculture and Forestry Office: 2008 – 2009  Annual Report of Thongmixai District Agriculture and Forestry Office: 2010 – 2011  Annual Report of Thongmixai District Agriculture and Forestry Office: 2011 – 2012 | Thongmixai DAFO  Thongmixai DAFO  Thongmixai DAFO  Thongmixai DAFO | Hardcopy  Hardcopy  Hardcopy  Hardcopy | Mar 2011  Mar 2011  Feb 2012  May 2012 |
| Agricultural statistics in Kentao 2006, 2008, 2010, 2011 | Summary of Socio-economic Performance of Kentao District 2007 – 2008  Summary of Socio-economic Performance of Kentao District 2008 – 2009  Annual Report of Kentao District Agriculture and Forestry Office: 2010 – 2011  Annual Report of Kentao District Agriculture and Forestry Office: 2011 – 2012 | Kentao DPI  Kentao DPI  Kentao DAFO  Kentao DAFO | Word file  Word file  Hardcopy  Hardcopy | Mar 2011  Mar 2011  Feb 2012  May 2012 |

| **Data** | **Source** | **Government unit** | **Obtained format** | **Period the data obtained** |
| --- | --- | --- | --- | --- |
| Agricultural statistics in Boten 2006, 2008, 2010, 2011 | 5-Year Summary of Boten District Agriculture and Forestry Office: 2006 – 2010  Annual Report of Boten District Agriculture and Forestry Office: 2011 – 2012 | Boten DAFO  Boten DAFO | Hardcopy  Hardcopy | Mar 2011  May 2012 |
| Agricultural area in Xayaburi Province 1976 – 2010 | National agriculture statistic year book 2012 | MAF | Book | May 2012 |
| Income and poverty statistics in Xayaburi and Xaysathan 2008 | Statistic year book of Xayaburi district 2008 | Xayaburi DPI | Hardcopy | Mar 2011 |
| Income and poverty statistics in Phiang 2008 | Statistic records 2008 – 2009 of Phiang district (from Phiang DPI database) | Phiang DPI | Excel file | Mar 2011 |
| Income and poverty statistics in Paklai 2008 | Annual statistic records of Paklai district 2008 (from Paklai socio-economic database)  Paklai District Socio-economic Plan: 2011 – 2015 | Paklai DPI  Paklai DPI | Hardcopy  Hardcopy | Mar 2011  Mar 2011 |
| Income and poverty statistics in Thongmixai 2008 | Statistic records 2008 – 2009 of Thongmixai district (from Thongmixai DPI database) | Thongmixai DPI | Hardcopy | Mar 2011 |
| Income and poverty statistics in Kentao 2008 | Statistic records 2008 – 2009 of Kentao district (from Kentao DPI database)  Summary of Socio-economic Performance of Kentao District of the year 2011 – 2012 and Direction for the Year 2012 – 2013 | Kentao DPI  Kentao DPI | Excel file  Hardcopy | Mar 2011  May 2012 |
| Income and poverty statistics in Boten 2008 | Statistic records 2008 – 2009 of Boten district (from Boten DPI database) | Boten DPI | Excel file | Mar 2011 |

**Abbreviation Note:**

PAFO: Provincial Agriculture and Forestry Office

DAFO: District Agriculture and Forestry Office

DPI: District Office of Planning and Investment

DOIC: District Office of Industry and Commerce

MAF: Ministry of Agriculture and Forestry
