# Supplementary material for: Balancing cash and food: The impacts of agrarian change on rural land use and wellbeing in Northern Laos
Source: PLoS One. 2018 Dec 31;13(12):e0209166. doi: 10.1371/journal.pone.0209166 (PMC6312269; doi:10.1371/journal.pone.0209166)
Supplement: S2 File — This file is the household interview form that is translated from the original version written in Laos. (DOC) [file pone.0209166.s002.doc]

**Household Interview Form**

Interviewee Name : ..........................................................

Village : ..........................................................

District :............................................................

Interviewer : ...........................................................

| **Interviewee’s Agreement**  I have been informed and acknowledged that this interview is part of the research “Effects of Market Integration on Land Use and Welfare in Xayaburi, Lao PDR” conducted by Mr. Puwadej Thanichanon from the Centre of Development and Environment (CDE), University of Bern, Switzerland. The research is taken under the collaboration between Department of Agriculture, Government of Lao and the Centre of Development and Environment (CDE), University of Bern, Switzerland.  I agree to participate in the interview and provide the data for the research. I understand that my personal data such as name, address and phone number will not be revealed to people outside the research and the data will be analyzed anonymously.  Name of Interviewee _________________________Signature___________ Date _________  Researcher _________________________________Signature___________ Date _________ |
| --- |

**1. Production data**

| **Crop** | **Area** | **Place to sell** | **Percent** | **Period** | **Quantity / yr** | **Price** |
| --- | --- | --- | --- | --- | --- | --- |
|  |  |  |  |  |  |  |
|  |  |  |  |  |  |  |
|  |  |  |  |  |  |  |
|  |  |  |  |  |  |  |
|  |  |  |  |  |  |  |

**2. Personal Data**

- Ethnicity / tribe ................................................................
- Education .................................................................
- No. of family members .................................................................
- Annual income …………………………………………..
- Vehicle .................................................................
- Information accessibility .................................................................
- Agricultural input / Self investment?
- Plowing ( Investor , Self , Own , No use ) ............................... Kips
- Seed ( Investor , Self , No use ) ............................... Kips
- Fertilizer ( Investor , Self , No use ) ............................... Kips
- Pesticide ( Investor , Self , No use ) ............................... Kips

**3. Problems**

- Price changes
- Quality & quantity of products
- Investor / company / merchant

...............................................................................................................................................................................................................................................................................................................................................................................................................................................................................................................................................................................................................................................................................

- What are your advantages or disadvantaged in trading opportunity? Are they related to wealthy, ethnicity, education?

.............................................................................................................................................................................................................................................................................................................................

**4. Opinion on market of your crops in the future**

...............................................................................................................................................................................................................................................................................................................................................................................................................................................................................................................

- How long you have grown the crop ...................................................
- The reason to grow this crop ……………………………………

…………………………………………………………………………………….……………………………………………………………………………………..

- Are you in interested in changing the crop species? ………………

……………………………………………………………………………………………………………………………………………………………………………………………………………………………….

**5. Questions of the changes**

- Land use ........................................................................................
- Infrastructure ......................................................................................
- Feeling (+ , - ) ..............................................................................
- Welfare ........................................................................................

......................................................................................................................................................................................................................................................................................................................................................................................................................................................................................................

**6. Buying of daily stuffs: place, vehicle, frequency**

- Grocery stores in the village ..............................................................
- Local market …………..............................................................
- Market 1 .......................................................................................
- Market 2 .......................................................................................

......................................................................................................................................................................................................................................................................................................................................................................................................................................................................................................
