# Supplementary material for: Balancing cash and food: The impacts of agrarian change on rural land use and wellbeing in Northern Laos
Source: PLoS One. 2018 Dec 31;13(12):e0209166. doi: 10.1371/journal.pone.0209166 (PMC6312269; doi:10.1371/journal.pone.0209166)
Supplement: S3 File — This file contains the study of land use change of the study area from satellite image analysis done by Boris Führer (unpublished work for Centre of Development and Environment, University of Bern, 2012) and document analysis from MAF and DAFOs. (DOCX) [file pone.0209166.s003.docx]

**Summary Results of Land Use Change**

**From Satellite Image Analysis and Document Analysis**

**Satellite Image Analysis**

The study of Boris Führer (unpublished work for Centre of Development and Environment, University of Bern, 2012) investigated changes in land cover in Xayaburi from 2001 to 2009 through GIS and remote sensing methods based on LANDSAT satellite images of 3 periods: (1) 05 / 01 / 2001, (2) 19 / 01 / 2006 and (3) 20 / 01 / 2009. These images were of the same season or month (January) but were taken in different years. The study classifies land cover into two types using biomass quantity detection as the classification criteria: (1) zero to low biomass area which is mostly supposed to be agricultural area, but possibly includes brush, urban, residential area and bare land, and (2) medium to high biomass area which is supposed to be forest and tree plantation. The change between the 2 types across the 3 periods is illustrated in the map in figure S1. The areas involving water bodies or covered by clouds, in even just one of three images, are excluded as unclassified areas. Table S1-1 explains the 8 types of land cover change in figure S.

**Table S3-1:** Description of land cover change between 2001, 2006 and 2009

| **Type** | **Color** | **Description** | **Area (km^2^)** | **%** |
| --- | --- | --- | --- | --- |
| 111 |  | Low biomass area since 2001 – existing agricultural area and urban area since 2001 | 586.0 | 5.2 |
| 112 |  | Area changed from low to high biomass after 2006 – crop area became forest or plantation area after 2006 or possible shifting cultivation area | 138.7 | 1.2 |
| 122 |  | Area changed from low to high biomass between 2001 and 2006 – crop area became forest or plantation area between 2001 and 2006 or shifting cultivation area | 131.4 | 1.2 |
| 121 |  | Area changed from low to high biomass between 2001 and 2006 and then back to low biomass after 2006 – possible shifting cultivation area | 109.9 | 1.0 |
| 212 |  | Area changed from high to low biomass between 2001 and 2006 and then back to high biomass after 2006 – shifting cultivation area or the area that had been cleared for farming but then was planted with trees again | 256.1 | 2.3 |
| 211 |  | Area changed from low to high biomass between 2001 and 2006 – forest area cleared for farmland between 2001 and 2006 | 197.4 | 1.7 |
| 221 |  | Area changed from high to low biomass after 2006 – forest area cleared for farmland after 2006 | 347.0 | 3.1 |
| 222 |  | High biomass area since 2001 – existing forest area since 2001 | 6,113.3 | 53.9 |

**Figure S3 Map of land cover change between 2001, 2006 and 2009**

**
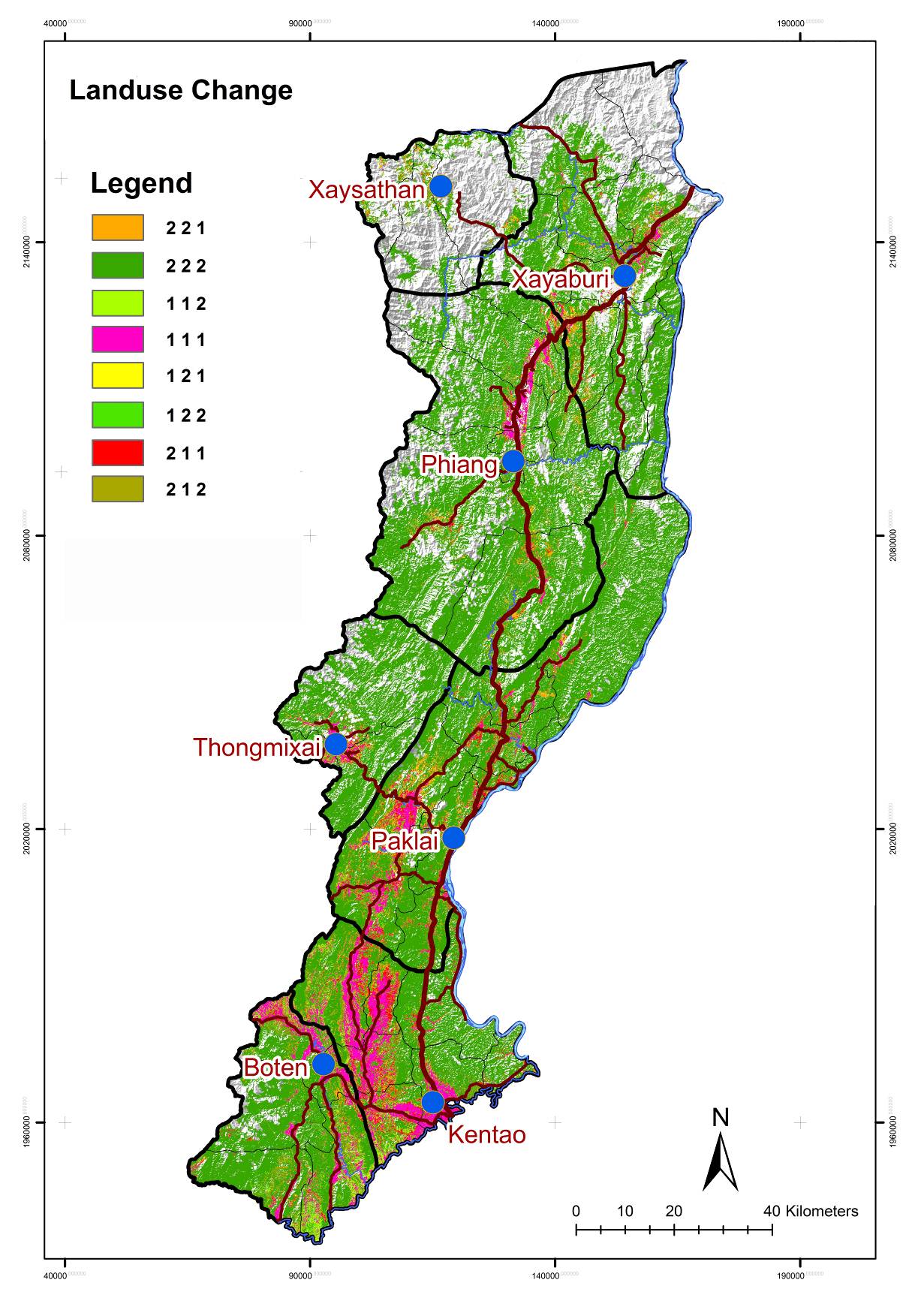
**

The results indicate that even though forest is still the most dominant land cover of the study area, a large amount of it was converted into agricultural area as there are high numbers of areas in type 211 (red) and 221 (orange) constituting 4.8 % of the study area. An area of at least 54,440 ha was converted from forest to farmland between 2001 and 2009. The period of 2006 – 2009 has a greater rate of agricultural expansion than the period of 2001 – 2006.

For considerations at the district level, table S1-2 provides the results of the expansion of agricultural area between 2001 – 2006 and 2006 – 2009, i.e. area of type 211 and 221 of each district based on the map in figure S1.

**Table S3-2:** Expansion of agricultural area between 2001, 2006 and 2009 at the district level

| **No** | **District** | **2001 – 2006** | **2006 – 2009** | **Total change** |
| --- | --- | --- | --- | --- |
| 1 | Xayaburi | 1,325.49 | 5,789.79 | 7,115.28 |
| 2 | Xaysathan | 294.59 | 1,417.73 | 1,712.32 |
| 3 | Phiang | 1,748.07 | 6,743.16 | 8,491.23 |
| 4 | Paklai | 6,528.25 | 11,082.44 | 17,610.69 |
| 5 | Thongmixai | 912.32 | 1,616.61 | 2,528.93 |
| 6 | Kentao | 7,372.20 | 5,957.05 | 13,329.25 |
| 7 | Boten | 1,830.12 | 2,090.38 | 3,920.50 |
|  | **Total** | **19,741.03** | **34,697.13** | **54,438.16** |

**Unit:** ha

The two major southern districts, Kentao and Paklai had the largest expansion of agriculture area in the period of 2001 – 2006. From 2006 to 2009, three northern districts: Xayaburi, Xaysathan and Phiang expanded much more than in previous years, while the southern districts had smaller expansion rates. Nevertheless, the amount of expanded agricultural area in Paklai and Kentao during the 2006 – 2009 period was greater than in other districts, thus reflecting that Paklai and Kentao are not only the areas where agricultural expansion began, but have also been the main agricultural zones for the entire study area.

The areas of type 112, 122 are 121 are relatively lower but the sum of these three types and type 212 is about 63,600 ha, which is relatively large and equal to 5.7 % of the study area. The areas possibly include shifting cultivation, or industrial plantations replacing former farmland, or deciduous forest. The area of type 212 in southern Boten is likely to be deciduous forest (as discussed in the next section).

**Limitations of interpretation from satellite image analysis**

Satellite image analysis through the remote sensing technique is a powerful way to detect the change in land cover. However, there are inevitable limitations which can lead to missing data, classification mistakes or errors in interpretation. The below points describe the main limitations.

1. A large amount of area is covered by cloud. This creates a high amount of unclassified area and thus a high percentage (30.1 %) of the ‘undetected’ classification, i.e. the area for which land cover change cannot be detected. Therefore, land cover classes of either forest or agricultural area are missing, as well as the change in those missing areas.

2. Each year has different natural conditions or weather conditions. Different moisture levels lead to wet and dry conditions and affects the quantity of biomass. This can leads to errors in interpretation of land cover type. For example, deciduous forest in very dry conditions may have very low biomass and can be wrongly interpreted as agricultural area. Irrigated paddy fields filled with water can also be wrongly interpreted as a body of water.

3. Some activities like extensive long-fallow shifting cultivation are highly dynamic or frequent changes to the land cover. Land cover under the shifting cultivation system is changed along the vegetation regeneration cycle from forest to farmland to brush land to shrub land. The period of this cycle may be short or long depending on various factors. This leads to difficulties in the interpretation of the land cover change, e.g. distinguishing whether the forest was cleared as new farmland or just temporarily cut for shifting cultivation, or whether agricultural land was replaced by tree plantations such as teak or rubber or just natural regrowth under shifting cultivation.

**Comparison of land use change between document analysis and satellite image analysis**

The comparison of land use change between document analysis and satellite image analysis was done in order to examine the precision of the analysis. Data from the land cover map of 2002 was compared with the satellite image of 2001. The data from the DAFOs in 2005 was compared with the satellite image of 2006. The data from the DAFOs in the year 2008 was compared with the satellite image from 2009. Table S1-3 compares the agricultural area from the document analysis with the satellite images at the district level presented above.

**Table S3-3:** Comparison of agricultural area between document statistics and satellite images in the years 2001/2002, 2005 and 2008

| **District** | **2001 / 2002** | | **2005** | | **2008** | |
| --- | --- | --- | --- | --- | --- | --- |
|  | Document | Satellite | Document | Satellite | Document | Satellite |
| Xayaburi | 67.24 | 139.40 | N/A | 92.20 | 142.24 | 169.83 |
| Xaysathan | 0.63 | 19.50 | N/A | 32.01 | 16.57 | 59.70 |
| Phiang | 59.77 | 110.57 | 85.37 | 106.00 | 111.54 | 178.96 |
| Paklai | 39.54 | 188.36 | 250.46 * | 249.74 | 331.82 | 336.83 |
| Thongmixai | 13.18 | 26.02 | 24.30 | 35.48 | 41.29 | 55.68 |
| Kentao | 37.28 | 319.43 | 147.00 | 440.71 | 255.84 | 387.88 |
| Boten | 58.62 | 221.98 | 50.33 | 284.63 | 84.00 | 171.60 |
| **Total** | **276.26** | **1,205.26** | **642.46 *** | **1,240.77** | **983.30** | **1,360.48** |

Unit: km^2^

Remark: * Estimated value

Figure below also illustrates the comparison of expansion of total agricultural area from document analysis and satellite image analysis. The results reveal the same trend of expansion of agricultural area converted from forest area. However, the amounts of area indicated by different sources were quite different across all three periods.

Agricultural area, as detected from satellite images in 2001, is much larger than indicated from the land cover map of 2002 in all districts. The low values in the land cover map of 2002 probably result from the classification criteria, which place other agricultural areas like temporary shifting cultivation into the unstocked forest and ray category, not agricultural area. Agricultural area from the satellite image of 2006 is also greater than that shown in the document statistics in 2005 in most of the districts. Considerably larger agricultural areas are reported in Kentao and Boten, though the area calculations are closer in Phiang, Paklai and Thongmixai.

The results from both sources show a sharper rate of agricultural expansion from 2005 to 2008 than in previous years. Document statistics reveal the sharper rate of expansion but the amount of area from satellite images is greater even since the beginning of the investigation in 2001. The difference in agricultural area is around 400 km^2^ in 2008, which is still rather high. This also implies that there are many unofficial agricultural lands in reality.
