# Supplementary material for: Balancing cash and food: The impacts of agrarian change on rural land use and wellbeing in Northern Laos
Source: PLoS One. 2018 Dec 31;13(12):e0209166. doi: 10.1371/journal.pone.0209166 (PMC6312269; doi:10.1371/journal.pone.0209166)
Supplement: S4 File — This file contains tables providing statistical data derived from government documents (MAF, Xayaburi PAFO and DAFOs) regarding land use change in Xayaburi province and the study area. (DOCX) [file pone.0209166.s004.docx]

**Supplementary Statistic Data**

**on Land Use and Land Use Change**

The statistic data shown on tables below are derived from government documents MAF, Xayaburi PAFO and DAFOs). The data regards area of different land use systems in Xayaburi province and the study area in different year and different districts to supplement the study on the part of “Expansion of cash crop cultivation” of the article.

**Table S4-1:** Agricultural area of main crops in Xayaburi province from 1976 to 2010

| **No** | **Crop** | **1976** | **1985** | **1995** | **2000** | **2002** | **2005** | **2010** |
| --- | --- | --- | --- | --- | --- | --- | --- | --- |
| 1 | Paddy field | 11,475 | 13,765 | 17,994 | 21,530 | 21,615 | 25,035 | 30,705 |
| 2 | Upland rice | 14,241 | 20,056 | 14,015 | 14,125 | 17,029 | 14,960 | 14,329 |
| 3 | Maize | 1,836 | 1,650 | 2,347 | 5,586 | 6,080 | 20,095 | 62,535 |
| 4 | Peanut | 137 | 280 | 1,512 | 2,439 | 2,662 | 2,320 | 3,700 |
| 5 | Cotton | 475 | 314 | 5,155 | 853 | 159 | 75 | 40 |
| 6 | Others | 1,600 | 4,445 | 14,557 | 11,049 | 7,477 | 3,878 | 8,125 |
|  | **Total** | **29,764** | **40,510** | **55,580** | **55,582** | **55,022** | **66,363** | **119,434** |

Unit: Hectare

Source of input data: MAF (2012)

**Table S4-2:** Change in area of main crops in the study area from 2006 to 2011

| **No** | **Crop** | **2006** | **2007** | **2008** | **2009** | **2010** |
| --- | --- | --- | --- | --- | --- | --- |
| 1 | Paddy field | 195.6 | 201.6 | 231.1 | 233.8 | 237.7 |
| 2 | Upland rice | 83.1 | 86.5 | 97.0 | 76.9 | 68.4 |
| 3 | Maize | 364.1 | 455.3 | 555.7 | 552.5 | 541.7 |
| 4 | Job’s tear | 13.8 | 13.1 | 21.4 | 24.9 | 65.0 |
| 5 | Other cash crops | 43.2 | 23.6 | 28.4 | 42.2 | 46.9 |
| 6 | Other crops | 7.5 | 19.9 | 49.7 | 43.7 | 24.4 |
|  | **Total** | **707.3** | **800.0** | **983.3** | **974.0** | **984.1** |

Source of input data: DAFOs (2012)

**Table S4-3(1):** Area of main agricultural land use systems at the district level in 2008

| **No** | **District** | **Paddy rice** | **Upland rice** | **Maize** | **Job’s tear** | **Cassava** | **Others** | **Total agri. Land** |
| --- | --- | --- | --- | --- | --- | --- | --- | --- |
| 1 | Xayaburi | 39.2 | 39.6 | 45.1 | 6.1 | 0.0 | 12.3 | **142.3** |
| 2 | Xaysathan | 0.4 | 14.6 | 0.0 | 0.0 | 0.0 | 1.6 | **16.6** |
| 3 | Phiang | 48.9 | 19.2 | 38.1 | 1.8 | 0.0 | 3.5 | **111.5** |
| 4 | Paklai | 56.6 | 8.6 | 245.4 | 3.3 | 0.0 | 17.9 | **331.8** |
| 5 | Thongmixai | 12.4 | 0.0 | 17.2 | 10.2 | 0.0 | 1.5 | **41.3** |
| 6 | Kentao | 45.6 | 11.0 | 182.0 | 0.0 | 0.0 | 17.2 | **255.8** |
| 7 | Boten | 28.0 | 4.0 | 27.9 | 0.0 | 0.0 | 24.2 | **84.0** |
|  | **TOTAL** | **231.1** | **97.0** | **555.7** | **21.4** | **0.0** | **78.1** | **983.3** |

Unit : km^2^

Source of input data: PAFO (2008), DAFOs (2008)

**Table S4-3(2):** Percentage of main agricultural land use systems at the district level in 2008

| **No** | **District** | **Paddy rice** | **Upland rice** | **Maize** | **Job’s tear** | **Cassava** | **Others** | **Total agri. Land** |
| --- | --- | --- | --- | --- | --- | --- | --- | --- |
| 1 | Xayaburi | 27 | 28 | 32 | 4 | 0 | **9** | **100** |
| 2 | Xaysathan | 2 | 88 | 0 | 0 | 0 | **10** | **100** |
| 3 | Phiang | 44 | 17 | 34 | 2 | 0 | **3** | **100** |
| 4 | Paklai | 17 | 3 | 74 | 1 | 0 | **5** | **100** |
| 5 | Thongmixai | 30 | 0 | 42 | 25 | 0 | **3** | **100** |
| 6 | Kentao | 18 | 4 | 71 | 0 | 0 | **7** | **100** |
| 7 | Boten | 33 | 5 | 33 | 0 | 0 | **29** | **100** |
|  | **TOTAL** | **23** | **10** | **57** | **2** | **0** | **8** | **100** |

**Table S4-4(1):** Area of main agricultural land use systems at the district level in 2011

| **No** | **District** | **Paddy rice** | **Upland rice** | **Maize** | **Job’s tear** | **Cassava** | **Others** | **Total agri. Land** |
| --- | --- | --- | --- | --- | --- | --- | --- | --- |
| 1 | Xayaburi | 40.9 | 31.8 | 42.0 | 15.9 | 0.0 | 5.1 | 135.7 |
| 2 | Xaysathan | 0.0 | 29.0 | 0.0 | 0.0 | 0.0 | 1.7 | 30.7 |
| 3 | Phiang | 55.3 | 11.3 | 28.9 | 21.1 | 0.0 | 3.1 | 119.7 |
| 4 | Paklai | 57.9 | 5.7 | 231.4 | 19.6 | 10.6 | 7.7 | 332.9 |
| 5 | Thongmixai | 12.7 | 0.3 | 8.8 | 14.4 | 0 | 0.5 | 36.7 |
| 6 | Kentao | 47.8 | 10.3 | 190.4 | 4.7 | 15.1 | 16.3 | 284.6 |
| 7 | Boten | 29.2 | 2.8 | 27.3 | 0.4 | 0.6 | 8.9 | 69.2 |
|  | **TOTAL** | **243.8** | **91.2** | **528.8** | **76.1** | **26.3** | **43.3** | **1009.5** |

Unit : km^2^

Source of input data: DAFOs (2012)

**Table S4-4(2):** Percentage of main agricultural land use systems at the district level in 2011

| **No** | **District** | **Paddy rice** | **Upland rice** | **Maize** | **Job’s tear** | **Cassava** | **Others** | **Total agri. Land** |
| --- | --- | --- | --- | --- | --- | --- | --- | --- |
| 1 | Xayaburi | 30 | 23 | 31 | 12 | 0 | 4 | **100** |
| 2 | Xaysathan | 0 | 94 | 0 | 0 | 0 | 6 | **100** |
| 3 | Phiang | 46 | 9 | 24 | 18 | 0 | 3 | **100** |
| 4 | Paklai | 17 | 2 | 70 | 6 | 3 | 2 | **100** |
| 5 | Thongmixai | 35 | 1 | 24 | 39 | 0 | 1 | **100** |
| 6 | Kentao | 17 | 4 | 67 | 2 | 5 | 5 | **100** |
| 7 | Boten | 42 | 4 | 39 | 1 | 1 | 13 | **100** |
|  | **TOTAL** | **24** | **9** | **52** | **8** | **3** | **4** | **100** |
