# Supplementary material for: Balancing cash and food: The impacts of agrarian change on rural land use and wellbeing in Northern Laos
Source: PLoS One. 2018 Dec 31;13(12):e0209166. doi: 10.1371/journal.pone.0209166 (PMC6312269; doi:10.1371/journal.pone.0209166)
Supplement: S6 File — The file explains the criteria for selecting sample villages for the household survey. It also contains a table that reveals the different characteristics of sample villages along the criteria and a figure that illustrates the locations of sample villages. (DOCX) [file pone.0209166.s006.docx]

**Selection of Sample Villages for the Household Survey**

A household survey of the study was conducted in order to obtain data about market access from the primary source at the finest level. The survey aimed to collect data from household samples throughout the study area in order to represent the region accurately. Therefore, 15 villages are selected as sample villages from which household samples were collected.

The selection criteria for sample villages mainly stresses geographic location and accessibility factors. These criteria include distance to town, to major border crossing, to minor border crossing, to Mekong River, to main road, elevation and landform. Table S6 reveals the different characteristics of sample villages among the criteria in a comparative manner and figure S6 illustrates the locations of 15 sample villages. It is seen from the table and the map that the 15 sample village locations are distributed rather regularly throughout the region and have some similarities and differences to one another, hence are good representatives for the region.

The study set the number of 15 sample villages for ease in recognition. The number started from the south to the north considering that the south has better market accessibility. The 15 sample villages were located in 15 different clusters and 7 districts in the study area. The numbers and names of the 15 sampled villages were 1) Na Hin, 2) Huay Lerk, 3) Mueng Pae, 4) Vern Kham, 5) Dong Sa Nguan, 6) Pa Lai, 7) Na Nhok, 8) Nam Lin, 9) Pha Liab, 10) Phone Sa Ad, 11) Som Sa Wan, 12) Nam Tuan, 13) Na Tak, 14) Pak Hung, and 15) Pak Long. Village 1 to 8 are regarded as villages in the south while village 9 to 15 are regarded as villages in north.

**Figure S6 Map of locations of sample villages.**

**The figure is based on public domain data from Openstreet map and was produced on QGIS 2.16**

**
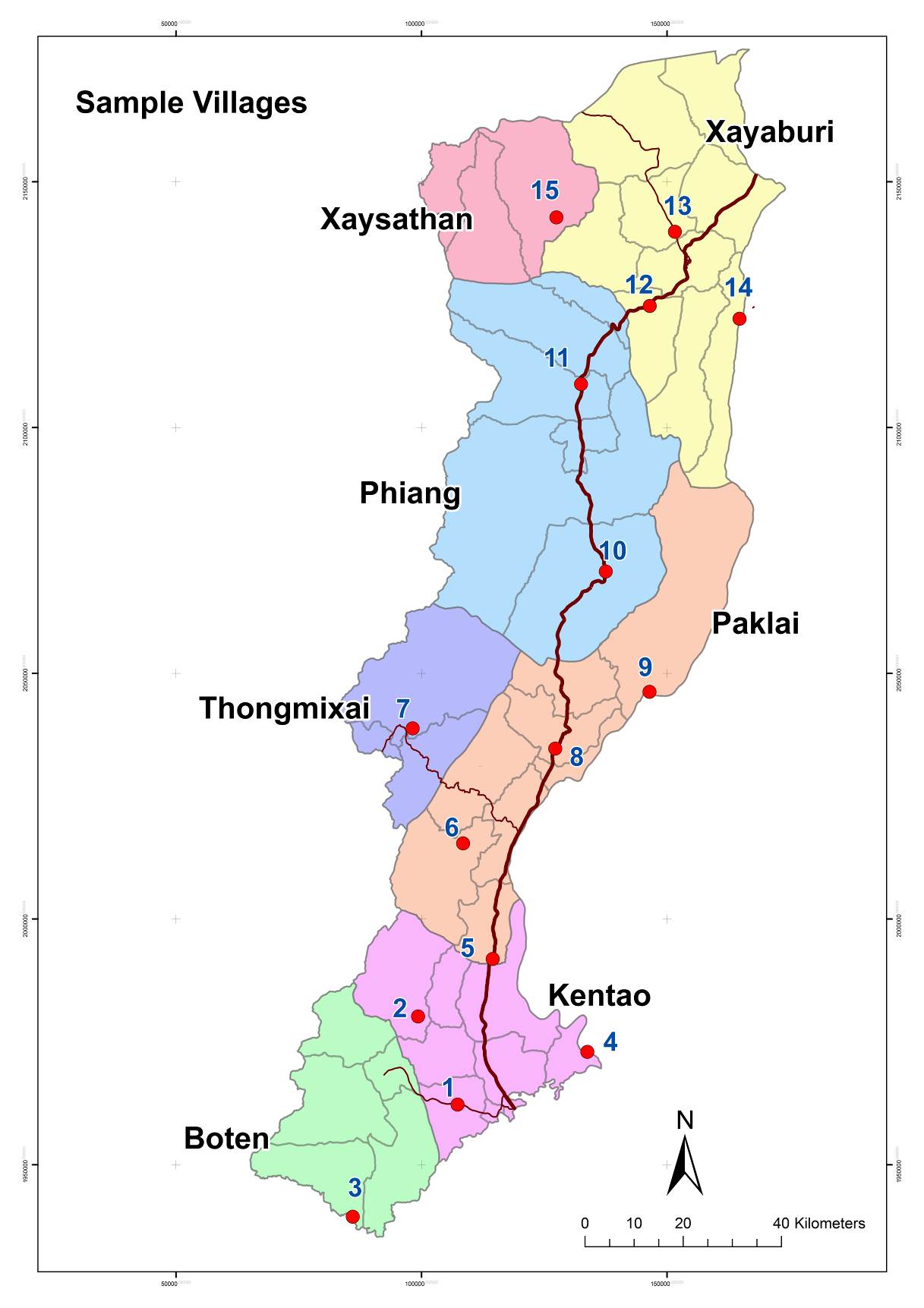
**

**Table S6:** Comparative characteristics of sample villages by the criteria of geographic location and accessibility factors

| **No** | **Village** | **Location** | **D. to town** | **D. to main road** | **D. to major crossing** | **D. to near crossing** | **D. to market** | **D. to Mekong** | **Landform** |
| --- | --- | --- | --- | --- | --- | --- | --- | --- | --- |
| 1 | Na Hin | South | Close | Close | Close | Close | Close | Far | Plain |
| 2 | Huay Lerk | South | Medium | Medium | Medium | Medium | Close | Very far | High hilly |
| 3 | Mueng Pae | South | Far | Far | Far | Close | Close | Very far | Hilly shore |
| 4 | Vern Kham | South | Far | Far | Medium | Close | Close | Close | Hilly shore |
| 5 | Dong Sa Nguan | South | Medium | Close | Close | Close | Medium | Medium | Low hilly |
| 6 | Pa Lai | Middle | Medium | Medium | Far | Medium | Medium | Medium | High hilly |
| 7 | Na Nhok | Middle | Close | Far | Very far | Close | Close | Far | Plateau |
| 8 | Nam Lin | Middle | Close | Close | Far | Far | Close | Medium | Low hilly |
| 9 | Pha Liab | Middle | Far | Far | Very far | Very far | Far | Close | Plain shore |
| 10 | Phone Sa Ad | Middle | Medium | Close | Very far | Very far | Close | Far | Plain |
| 11 | Som Sa Wan | North | Close | Close | Very far | Very far | Close | Far | Plain |
| 12 | Nam Tuan | North | Close | Close | Very far | Very far | Close | Medium | Low hilly |
| 13 | Na Tak | North | Close | Close | Very far | Very far | Close | Medium | Low hilly |
| 14 | Pak Hung | North | Medium | Medium | Very far | Very far | Far | Close | Hilly shore |
| 15 | Pak Long | North | Far | Far | Very far | Very far | Far | Very far | Mountain |
